# Mining methods and typical structural mechanisms of terpene cyclases

**DOI:** 10.1186/s40643-021-00421-2

**Published:** 2021-07-28

**Authors:** Zheng-Yu Huang, Ru-Yi Ye, Hui-Lei Yu, Ai-Tao Li, Jian-He Xu

**Affiliations:** 1 State Key Laboratory of Bioreactor Engineering, Shanghai Collaborative Innovation Centre for Biomanufacturing, Frontiers Science Center for Materiobiology and Dynamic Chemistry, East China University of Science and Technology, Shanghai, 200237 China; 2 State Key Laboratory of Biocatalysis and Enzyme Engineering, Hubei Collaborative Innovation Center for Green Transformation of Bio-Resources, Hubei Key Laboratory of Industrial Biotechnology, School of Life Sciences, Hubei University, Wuhan, 430062 China

**Keywords:** Terpene cyclases, Biochemical properties, Review, Genomic mining, Structural analysis, Catalytic mechanisms

## Abstract

Terpenoids, formed by cyclization and/or permutation of isoprenes, are the most diverse and abundant class of natural products with a broad range of significant functions. One family of the critical enzymes involved in terpenoid biosynthesis is terpene cyclases (TCs), also known as terpene synthases (TSs), which are responsible for forming the ring structure as a backbone of functionally diverse terpenoids. With the recent advances in biotechnology, the researches on terpene cyclases have gradually shifted from the genomic mining of novel enzyme resources to the analysis of their structures and mechanisms. In this review, we summarize both the new methods for genomic mining and the structural mechanisms of some typical terpene cyclases, which are helpful for the discovery, engineering and application of more and new TCs.

## Introduction

### Biosynthesis of terpenoids

Terpenoids are one of the most diverse natural compounds which include terpenes with a number of isoprene (C5) units, as well as C5 polymers containing phosphate, hydroxyl, carboxyl, aldehyde and other functional groups (Dickschat 2016; Helfrich et al. 2019). According to the data currently recorded in the Dictionary of Natural Products (http://dnp.chemnetbase.com), the number of terpenoids has reached 80,000 (Chen et al. 2020a). These terpenoids play important roles in higher plants, fungi, bacteria, insects and marine organisms, such as plant hormones; carotenoids in photosynthesis; steroids in cell membrane; quinone compounds transferring electron (Gao et al., 2012). In addition to the daily used perfumes, resins and pigments, terpenoids have a broader application prospect in the field of medicine. A typical example is paclitaxel (e.g., Taxol®), a diterpenoid compound isolated from the endophytic fungus of *Taxus brevifolia* or the bark of the *Pacific yew* (*T. brevifolia*). Due to the unique anti-cancer mechanism and excellent efficacy, paclitaxel is widely used for the treatments of breast cancer, soft nest cancer and lung cancer, among others (Miele et al. 2012). In recent years, more functions of terpenoids have been reported, such as the treatment of diabetes by inhibiting the activity of α-glycosidase (Valdes et al. 2020), and overcoming the multidrug resistance in tumor treatment as ABC transporter modulators (Goncalves et al. 2020), etc. These achievements indicate the broad application prospects of terpenoids, which in turn stimulates researchers' interest in the biosynthetic pathways of terpenoids.

Terpenoids are originated from two common precursor substances: isopentenyl diphosphate (IPP) and dimethylallyl diphosphate (DMAPP). The synthesis of IPP and DMAPP involves two different pathways: in most prokaryotes and plant plastids, these compounds are produced through the 2-methyl-d-erythritol-4-phosphate (MEP) pathway; In most eukaryotes, archaea and some prokaryotes, they are produced by mevalonate acid (MVA) pathway (Daletos et al. 2020). IPP can be isomerized to DMAPP by isopentenyl pyrophosphate isomerase (Hampel et al. 2005).

**Fig. 1:**
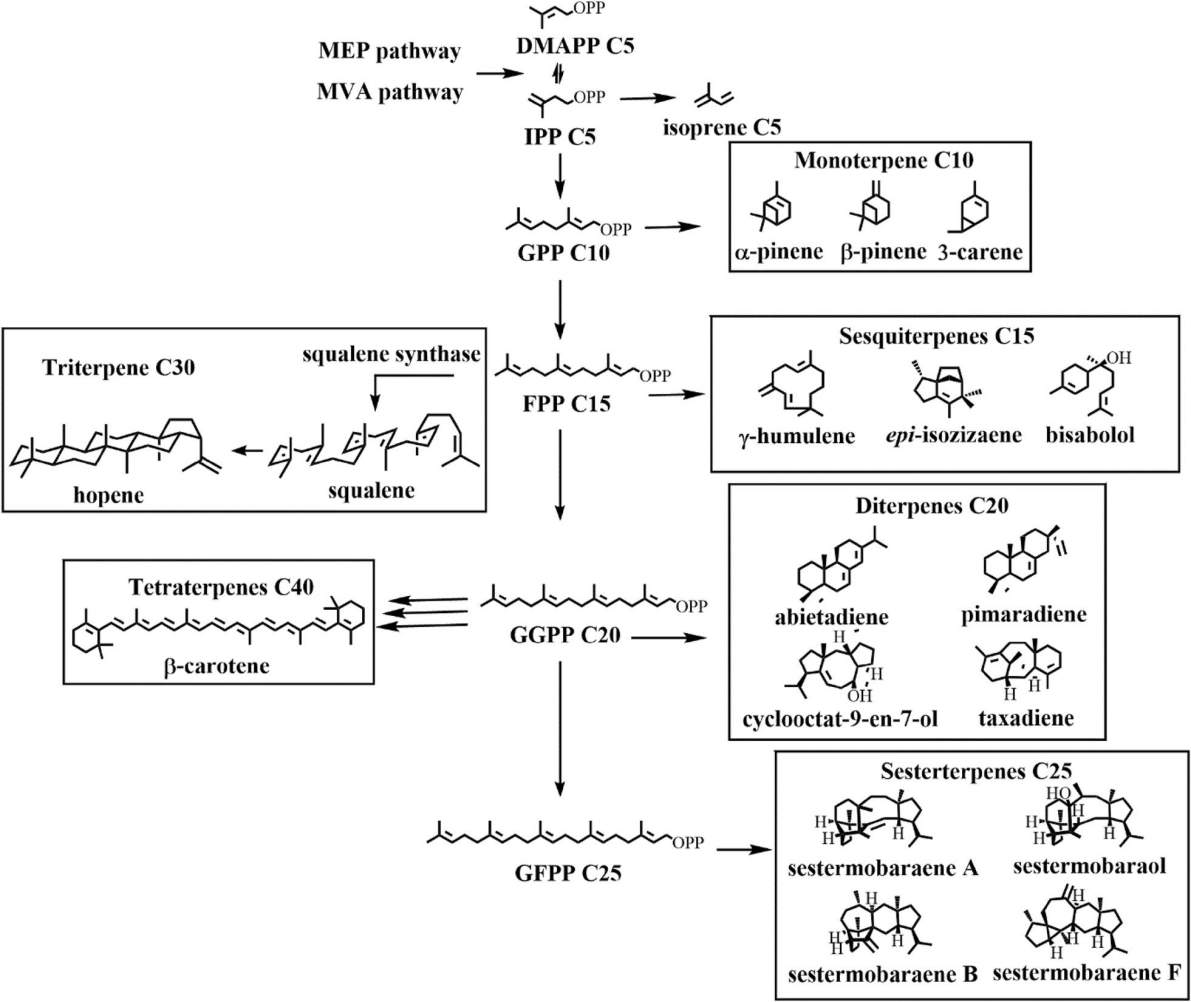
Biosynthesis pathway of different terpenoids. OPP: pyrophosphate group

IPP and DMAPP, as active structural units, are cyclized or arranged to form isoprenoid oligomers that conform to ‘C5’ or ‘biogenetic isoprene’ rule (Ruzicka 1953). These oligomers are the direct precursors of terpenoids, such as geranyl pyrophosphate (GPP), farnesyl pyrophosphate (FPP) and geranylgeranyl pyrophosphate (GGPP). After the formation of GPP, FPP and GGPP, various terpenes were formed under the catalysis of terpene cyclases (TCs). According to the number of C_5_H_8_ units, terpenes can be classified into hemiterpenes (C5), monoterpenes (C10), sesquiterpenes (C15), diterpenes (C20), sesterterpenes (C25), triterpenes (C30), tetraterpenes (C40), etc. (Fig. 1).

Monoterpene (C_10_H_16_) is formed by two isoprene units (cyclic or non-cyclic). Common monoterpenes and monoterpenoids are typical volatile compounds that can be isolated from essential oils of various herbs and citrus fruits (Davis and Croteau 2000). Sesquiterpene (C_15_H_24_) is composed of three isoprene units and usually found in essential oils and extracts, including santalol, caryophyllene, and humic acid. Diterpene (C_20_H_32_) is formed by four isoprene units. In general, diterpenoids have a ring structure and are subsequently oxidized into alcohols, aldehydes or acids (Keeling and Bohlmann 2006), such as diterpene resin acids which are abundant in conifers. Triterpenes (C_30_H_48_), composed of six isoprene units, are a class of compounds including squalene and sterol precursors. Tetraterpenes (C_40_H_64_), composed of eight isoprene units, are mainly carotene and carotenoid compounds. The higher polyisoprene compounds are the structural basis of natural rubber and latex.

### The catalytic mechanism and classification of terpene cyclases

Terpenoids have both simple linear hydrocarbon chain structures and complicated cyclic structures. The complex structure of terpenoids is formed by the cyclization of linear, chiral polyisoprene substrates. In terpenoid biosynthesis, cyclization is usually the first step of the synthesis process, followed by hydroxylation or other modifications to produce terpene hydrocarbons or terpene alcohols. This vital cyclization reaction is catalyzed by TCs. Interestingly, some TCs may have multiple active centers, thereby completing the construction of multiple cyclization and chiral centers in a single-step reaction (Li et al. 2020). For cyclases, a small change in the protein structure may result in great impacts on its activity, and changes in some key sites may produce brand-new catalytic activity (Keeling et al. 2008).

**Fig. 2:**
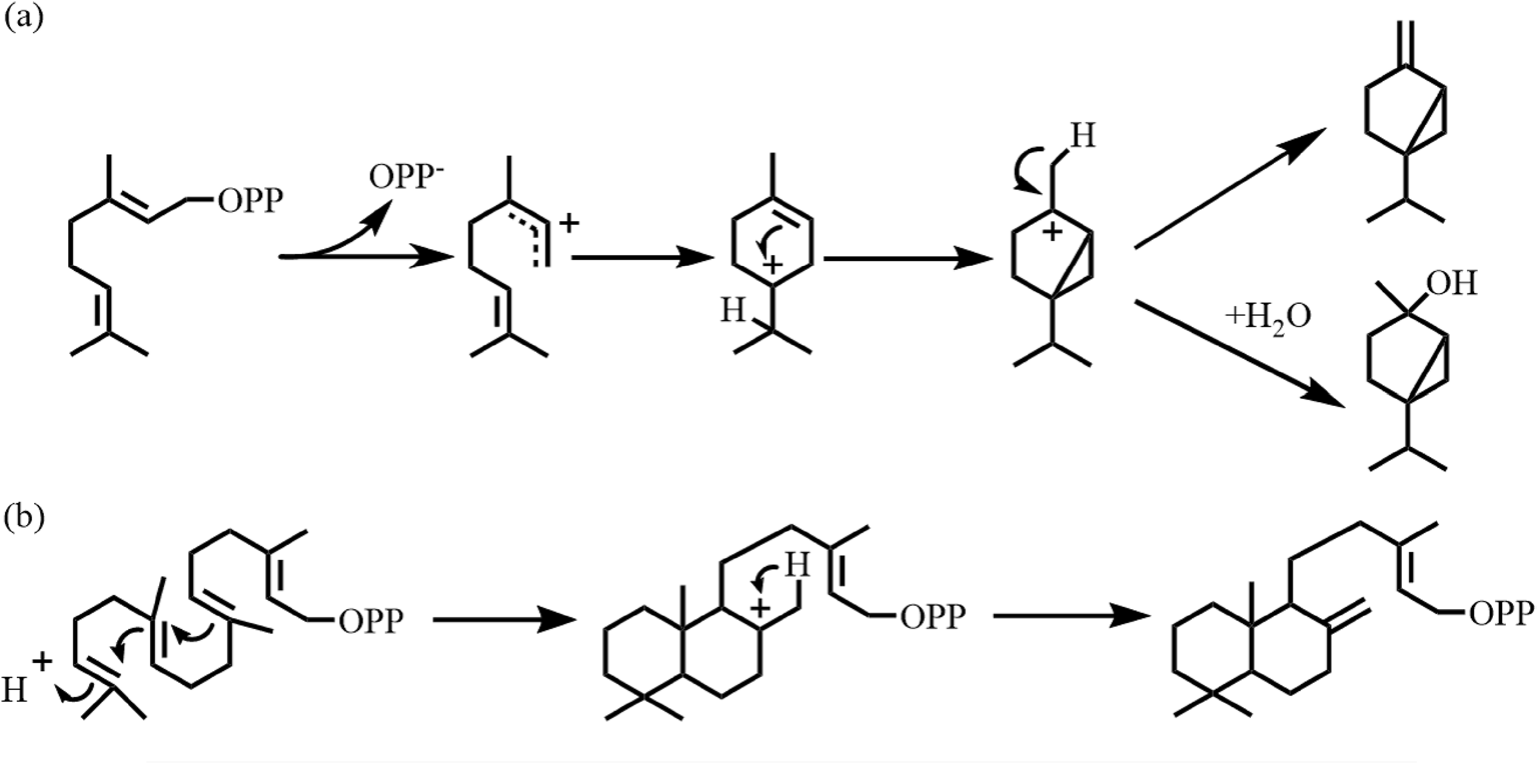
Different formation mechanisms of carbocation in class I and class II terpene cyclases. **a** Ionization-dependent reaction catalyzed by class I terpene cyclases with GPP as the substrate. **b** Protonation-dependent reaction catalyzed by class II terpene cyclases with GGPP as the substrate

TCs-catalyzed reaction usually starts with the formation of carbocations, so TCs can be classified according to the formation mechanism of carbocations (Wendt and Schulz 1998): ionization-dependent TCs (class I, Fig. 2a) and protonation-dependent TCs (class II, Fig. 2b). Class I terpene cyclase initiates the cyclization reaction by ionization of the phosphate group. There is a conserved motif ‘DDXXD’ rich in aspartic acid in its active center, which combines with divalent metal ions (e.g., Mg^2+^), and attacks the pyrophosphate group of substrates through ionization of metal cations, then promotes the departure of pyrophosphate group and cyclization of linear molecules. Whereas class II terpene cyclase does not have the above-mentioned conserved motif rich in aspartic acid residues, but initiates the protonation ring reaction through the N-terminal conserved motif ‘DXDD’ (Dickschat 2016).

Although, terpene cyclases have relatively low *K*_M_ values, the extremely low *k*_cat_ values of terpene cyclases severely limit the efficient production of terpenes (Table 1). Understanding the structure and catalytic mechanism of terpene cyclase is the key to solve this problem.

**Table 1:** Kinetic parameters of terpene cyclases reported

Enzyme	Sources	*K*_M_ (μM)	*k*_cat_ (s^−1^)	Refs.
BPPS	*Salvia officinalis*	1.4	–	Croteau and Karp (1979)
(+)-LS	*Citrus sinensis*	13.1	–	Morehouse et al. (2017)
CS	*Salvia fruticosa*	65.4	0.053	Kampranis et al. (2007)
EBOS	*Lippia dulcis*	4.8	0.04	Attia et al. (2012)
EIZS	*Streptomyces coelicolor*	0.7	0.045	Aaron et al. (2010)
AS	*Penicillium roqueforti*	0.53	0.084	Faraldos et al. (2012)
EAS	*Nicotiana tabacum*	2.3	0.005	Rising et al. (2000)
TS	*Taxus brevifolia*	3.0	0.011	Williams et al. (2000)
CPPS	*Arabidopsis thaliana*	3.0	0.9	Koksal et al. (2014)

*BPPS* bornyl diphosphate synthase, *(+)-LS* (+)-limonene synthase, *CS* 1,8-cineole synthase, *EBOS* (+)-*epi*-α-bisabolol synthase, *EIZS* *epi*-isozizaene synthase, *AS* aristolochene synthase, *EAS* 5-*epi*-aristolochene synthase, *TS* taxadiene synthase, *CPPS* *ent*-copalyl diphosphate synthase

## Methods for discovering new terpene cyclases

Boutanaev et al. (2015) investigate the basis of terpenoid-scaffold diversity through analysis of multiple sequenced plant genomes and discovered that the primary drivers of terpenoid-scaffold diversity are terpene cyclases. At the beginning, researchers usually extract from plants or use degenerate primers for PCR to obtain terpene cyclases (Hezari et al. 1995; Kawaide et al. 1997), With the rapid development of high-throughput sequencing technology, microbial whole gene composition become an important source of terpene cyclases for researchers (Hou and Dickschat 2020; Li et al. 2020; Yang et al. 2017). Here we summarize several common methods of terpene cyclases mining to enhance the understanding of the methods of mining terpene cyclases and discover new terpene cyclases.

### Direct cloning

**Fig. 3:**
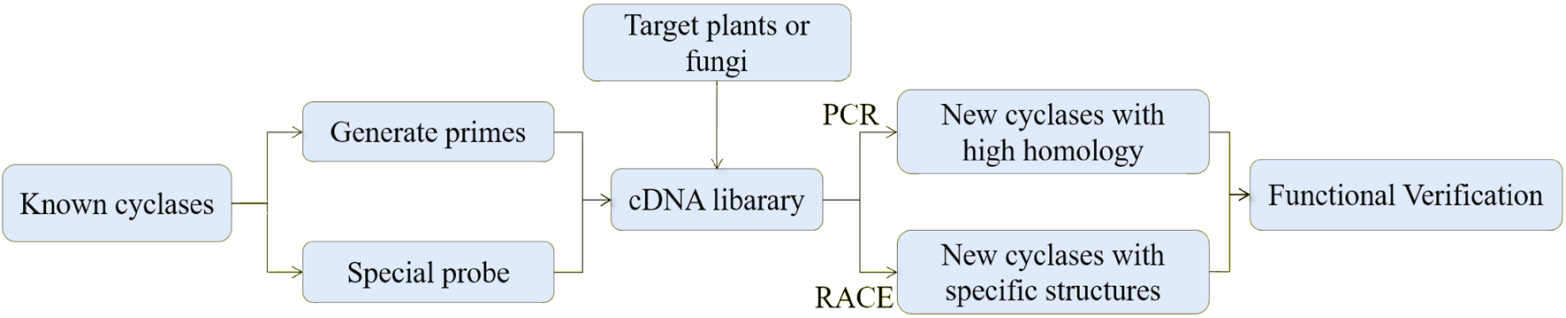
Direct cloning to discover new terpene synthases

In this method, degenerate primers are designed based on the highly conserved sequences obtained by homology analysis. The sequence fragments of related enzymes are amplified by PCR, and then the target enzyme genes are cloned by constructing a cDNA library (Fig. 3).

Kawaide et al. (1997) cloned the first fungal diterpene cyclase gene from the cDNA library of *Phaeosphaeria* sp. L487 by designing primers based on the conservative sequence of copalyl diphosphate (CPP) synthase in plants and amplifying the gene through PCR. The diterpene cyclase genes from *Gibberela fujikuroi* (Toyomasu et al. 2000) and *Phoma betae* (Oikawa et al. 2001) were also successfully cloned in the same way. Basyuni et al. (2007) cloned four multifunctional triterpene cyclases from *Bruguiera gymnorrhiza* leaves and *Rhizophora stylosa* using the homologous cloning method and successfully verified the substrates.

The cloning method based on sequence homology is direct and convenient, and is usually regarded as the first choice in gene cloning, but there are still certain limitations in the application. For example, when the enzymes are of low homology or have specific structures, the direct cloning method is often not suitable. For smaller diterpene cyclases, in addition to using probes for gene fishing in the cDNA library, the full length of target gene can also be obtained by first cloning the core fragments in conserved sequences and then extending to the complete sequence using the RACE (rapid amplification of cDNA ends) technology. For instance, Ye et al. (2018) used reverse transcription-polymerase chain reaction RT-PCR (reverse transcription-polymerase chain reaction) and RACE technology to clone a gene As-Ses TPS (sesquiterpene cyclases from *Aquilaria sinensis*) from the total RNA of *A. sinensis*. As-Ses TPS encodes a germacrene-d-synthetase which is located in the cytoplasm and only expressed in the scent-forming part of *A. sinensis*.

### Genome analysis

#### Genome sequencing

**Fig. 4:**
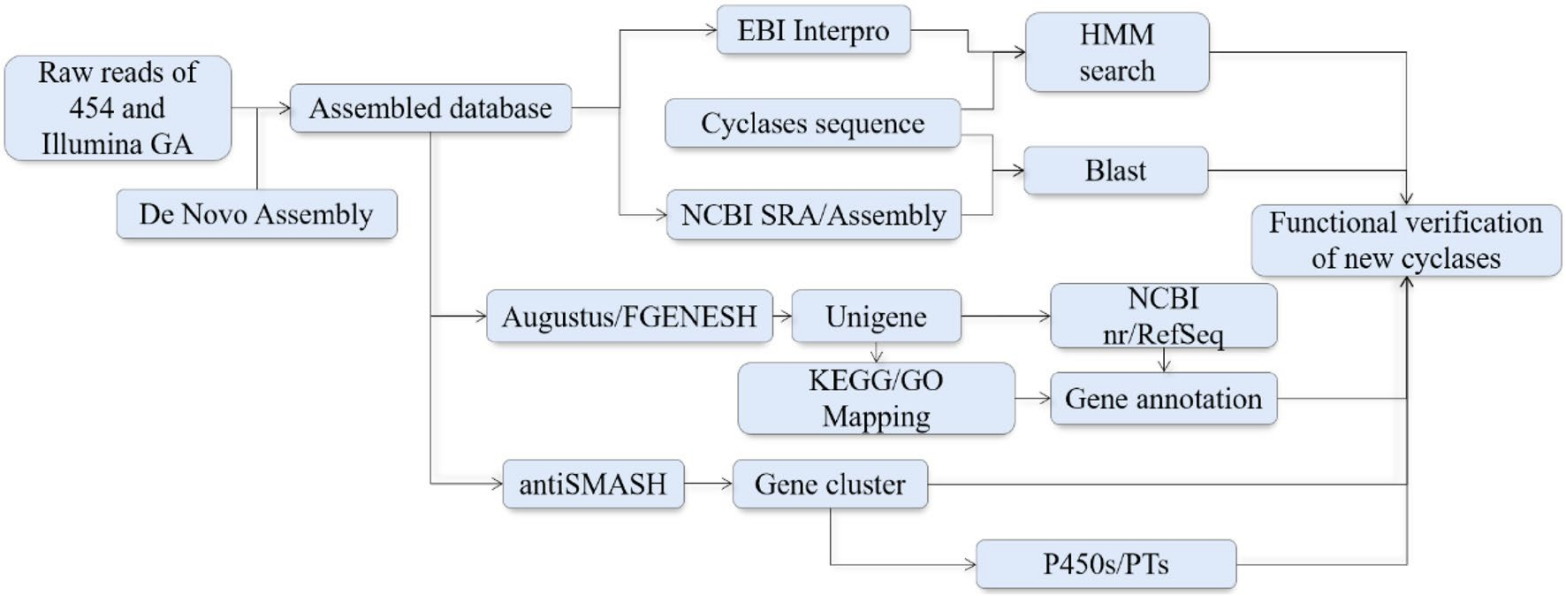
Genome analysis to discover new terpene synthases. *PTs* prenyltransferase

With the rapid development of DNA sequencing technology, the genomes of many species in addition to yeast, *Escherichia coli*, mice and other model organisms, have been sequenced and annotated. To obtain more comprehensive candidate genes in the genome, many databases have predicted the putative coding sequence while recording the genome information. For example, 362 fungal genomes are listed in the Joint Genomics Institute (JGI, http://genome.jgi-psf.org), which has the goal to gather 1000 sequenced genomes from all fungal families in the next few years. Through similar websites, genomes of target species and their gene annotation can be obtained (Fig. 4).

Shibuya et al. (2009) predicted 13 homologous genes of oxidosqualene cyclases (OSCs) in the *Arabidopsis* genome sequencing results, and obtained their corresponding cDNA by RT-PCR. The screening, expression and substrate validation had been done in *Saccharomyces cerevisiae* deficient in lanosterol synthase. Matsuda et al. (2015) sequenced the genome of *Emericella variecolor*, which can produce a variety of terpenoids. The selected candidate genes were chosen by domain prediction, domain and conservative sequence alignment methods. A number of terpene cyclases were successfully identified, and the closely related P450 monooxygenases in the same biosynthetic pathway were found from the gene clusters of these terpene cyclases.

#### Gene cluster retrieval

In the genomes of fungi and plants, the genes responsible for some secondary metabolites biosynthesis, such as polyketides, non-ribosomal polypeptides and terpenoids often gather in a form of clusters (Bills and Gloer 2016). Therefore, gene cluster retrieval is a useful method to discover new and novel enzymes in a certain biosynthesis pathway by mining and characterizing the adjacent genes according to the location of a related gene. This method has been widely used in gene mining of terpene cyclases from eukaryotic (especially fungal) sources (Fig. 4).

GGPP synthase is an essential enzyme for the synthesis of diterpene compounds in fungi. It is usually located on the biosynthetic gene cluster of diterpenes. Therefore, by cloning the GGPPs gene and analyzing the gene clusters, the adjacent diterpene cyclase genes can be obtained. Oikawa et al. (2001) employed this method to excavate the diterpene cyclase of aphidicolin in *P. betae*. Toyomasu et al. (2008) applied this method to excavation of the terpene cyclase from *Phomopsis amygdali*, and obtained the diterpene cyclase of labdanate. Compared with the direct gene cloning, the gene cluster retrieval method does not require the construction of cDNA library, nor does it rely on the homology of cyclase genes. It is an effective strategy for discovering novel terpene cyclase genes.

### Gene screening based on biosynthesis pathway

In *E. coli* with the MVA pathway, accumulation of IPP would be toxic to the bacterium and inhibit the growth of cells. If the genes that encode the IPP-consuming and terpene-synthesizing enzymes are introduced into the engineered strain, the growth inhibition might be relieved. For instance, Withers et al. (2007) introduced FPP synthase and sesquiterpene cyclase into *E. coli*, screened the cDNA library in a high-throughput mode, then obtained the gene encoding a hemiterpene cyclase that can relieve the growth inhibition. As a new way to find terpene cyclases, the gene screening approach based on biosynthesis pathway does not depend on sequence homology and is suitable for searching the terpene cyclase genes with unknown genetic information.

Another screening method based on biosynthesis pathway is to monitor the expression level of target proteins and the related terpenoid yield after adding inducer. Xu et al. (2013) cloned three full-length cDNAs (ASS1, ASS2, ASS3) of sesquiterpene cyclases which may be related to the formation of aroma from the library of *A. sinensis*, and expressed them in *E. coli*. After treated with a plant hormone, methyl jasmonate (MeJA), the expression of ASSs was significantly induced, and the corresponding yield of sesquiterpenes was also increased accordingly, so that the sesquiterpene cyclase with FPP as the substrate was successfully revealed.

### Transcriptome analysis

The expression of terpenoids will increase after the inducer is added, and the candidate genes of the relevant enzymes in the biosynthetic pathway of the target compound can be obtained by analyzing the changes in the transcriptome information before and after adding the inducer (Lange and Ahkami 2013). While mining new enzymes, this method can also provide a more complete understanding of the biosynthetic pathway of terpenoids.

Misra et al. (2014) detected the gene transcription changes of sweet basil treated with the secondary metabolic initiator MeJA and identified 388 candidate unique transcripts of MeJA responsiveness. Transcript analysis indicated that, in addition to controlling its own biosynthesis and stress response, MeJA also up-regulates the transcripts of various secondary metabolic pathways, including those of terpenoids and phenylpropane/flavonoids. In addition, the combination of transcript and metabolite analysis revealed the biosynthesis of medically significant urane-type and olean-type pentacyclic triterpenes induced by MeJA. By transcript analysis, two MeJA-responsive oxidiosqualene cyclases (ObAS1 and ObAS2) were successfully identified, which are composed of 761 and 765 amino acid residues, respectively.

## Class I terpene cyclases

Class I terpene cyclases contain DDXXD and NSE metal-binding motifs which are found with α, αβ and αβγ domain architectures (Gennadios et al. 2009; Janke et al. 2014; Koksal et al. 2011; Whittington et al. 2002). Class I terpene cyclase initiates the cyclization reaction by ionization of the phosphate group. Here, we introduce structure and cyclization mechanism of some class I terpene cyclases to enhance the acknowledge of class I terpene cyclases.

### Monoterpene cyclases

#### Bornyl diphosphate synthase (BPPS)

*So*BPPS, from *Salvia officinalis*, is the first monoterpene cyclase (PDB ID: **1N1B**) that obtained the crystal structure (Whittington et al. 2002). *So*BPPS is cyclized with GPP to produce (+)-bornyl diphosphate (BPP). Then BPP is dephosphorylated by phosphatase to generate borneol, which has anti-inflammatory, anti-oxidant, and enhanced GABA_A_ receptor functions (Granger et al. 2005; Zhang et al. 2017).

**Fig. 5:**
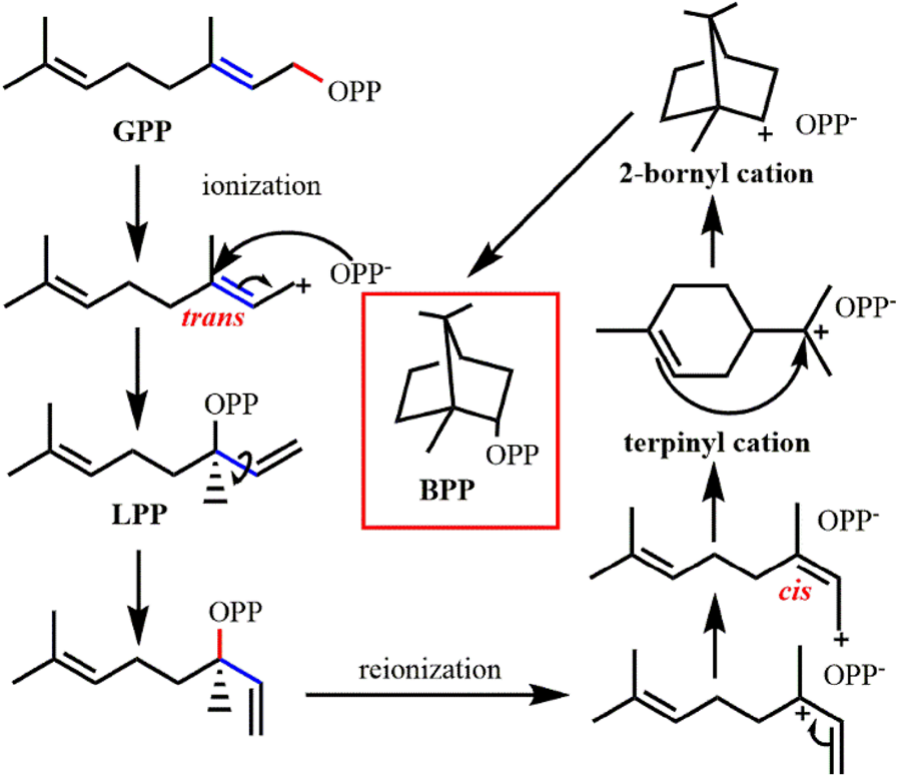
Cyclization mechanism of bornyl diphosphate synthase. *GPP* geranyl diphosphate, *LPP* (3*R*)-Linalyl diphosphate

The first reaction (Whittington et al. 2002) catalyzed by *So*BPPS is the formation of allyl carbocationic intermediate by the ionization of pyrophosphate group (Fig. 5), and then the pyrophosphate group is reassembled to C3 atom, forming (3*R*)-linalyl diphosphate (LPP). When the C2–C3 bond rotates to *cis*-configuration, the pyrophosphate group is ionized again to carry out the subsequent cyclization process (Fig. 5). The C2–C3 bond changes from *trans*-configuration to *cis*-configuration of substrate. This process is common in terpene cyclase (Hyatt et al. 2007; Kampranis et al. 2007; Morehouse et al. 2017; Rudolph et al. 2016), while there are also some cyclases that do not need configuration transformation, such as selinadiene synthase, whose C2–C3 bond still maintains *trans*-configuration (Baer et al. 2014).

**Fig. 6:**
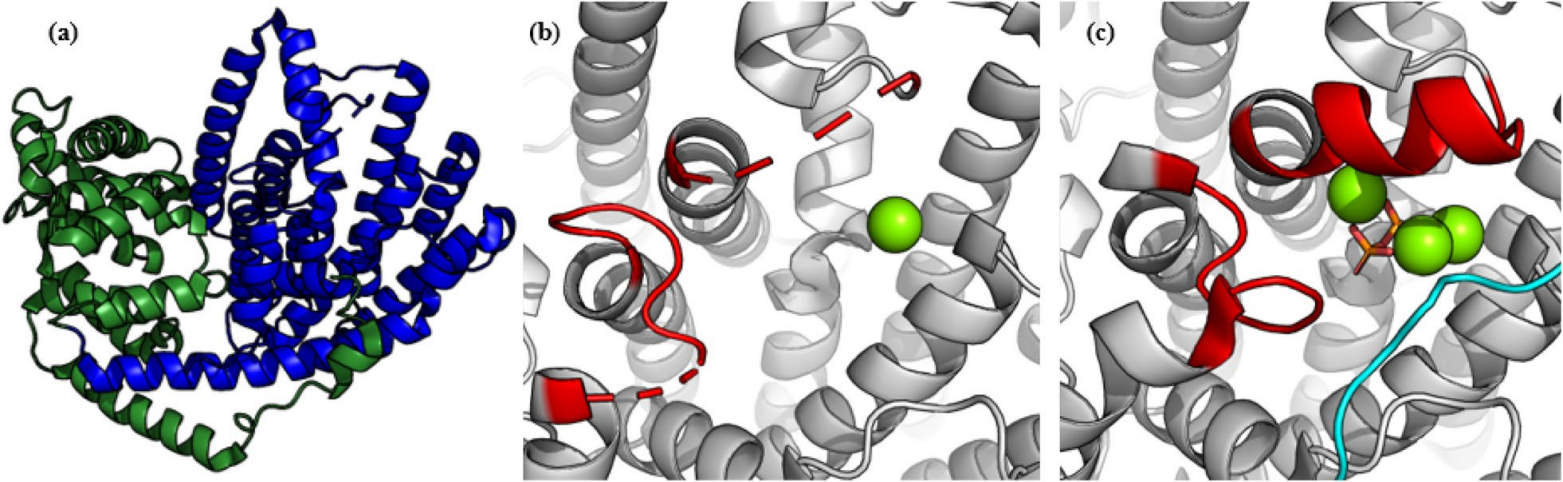
Structure of bornyl diphosphate synthase. **a** Stereoplot of unliganded (+)-bornyl diphosphate synthase (PDB ID: **1N1B**), looking into the active site in the α domain (blue). Disordered polypeptide segments are indicated by dashed lines and the β domain is in green. **b** Active site of unliganded (+)-bornyl diphosphate synthase. **c** Active site of the (+)-bornyl diphosphate synthase complexed with inorganic pyrophosphate and Mg^2+^ (green ball) (PDB ID: **1N1Z**). These conformational differences between unliganded enzyme and liganded enzyme mainly include the N-terminal segment (cyan) and some loops (red) which help ‘close’ the active site

BPPS (Fig. 6a) exists as a dimer (βα:αβ), whose active site is located in the α domain. It has the characteristic sequence of class I cyclase DDXXD and (N,D)DXX(S,T)XXXE. After binding with three Mg^2+^ and pyrophosphate groups, the conformation of BPPS changes from an open state to a closed state (Fig. 6b, c), to remove solvent and prevent carbon cation from being eliminated prematurely by solvent molecules during cyclization. F578 and W323 stabilize the reaction intermediates through cation–π interaction. In the closed conformation, water molecule #110 forms hydrogen bonds with Y426, S451 and pyrophosphate groups, which plays an important role in controlling the molecular conformation (Whittington et al. 2002). The three basic residues, R314, R493 and K512, help Mg^2+^ to better combine with pyrophosphate group and form the recognition motif of pyrophosphate with metal ions (Aaron and Christianson 2010).

Despinasse et al. (2017) isolated a new borneol pyrophosphate synthase (*La*BPPS) from *Lavandula angustifolia*, which possesses 50% identity to the protein sequence of BPPS originally isolated from *S. officinalis* (*So*BPPS). *La*BPPS produces more by-products, such as α-pinene, β-pinene, camphene, linalool and so on. By comparing the differences of sequences and structures between *La*BPPS and *So*BPPS, the key amino acid residues which can change product distribution or even the molecular structure of the product may be found. In recent years, computer technology has also been applied to the study of terpene cyclases. Methods, such as TerDockin (O'Brien et al. 2018) and EnzyDock (Das et al. 2019), can predict the orientation of substrates and carbocation intermediates, showing the importance of computer technology in the study of terpene cyclase structures.

#### Limonene synthase (LS)

Limonene is a class of chiral monoterpene molecules with two enantiomers (4*R*)-(+)-limonene and (4*S*)-(−)-limonene. Limonene has biofuel potential (Beller et al. 2015), antibacterial and antioxidant capabilities (Fahim et al. 2019) and cancer prevention effect. Some oral limonene and other limonene drugs for the treatment of breast cancer are in clinical trials (Silva et al. 2019; Singh and Sharma 2015).

(+)-Limonene and (−)-limonene are synthesized by specific limonene synthases. Hyatt et al. (2007) obtained the (−)-LS crystal structure (PDB ID: **2ONG** & **2ONH**) derived from *Mentha spicata*. Morehouse et al. (2017) obtained the (+)-LS crystal structure (PDB ID: **5UV0**) derived from *Citrus sinensis*. Similar to BPPS, LS has two domains (α & β). The catalytically active site of LS is located in α domain. Although the identity between the two LS sequences is only 44.7%, their amino acid residues in the active sites are highly conserved (Morehouse et al., 2017).

Kumar et al. (2017) examined the reasons of the two LSs for generating stereoselectivity, and found that the residues M458/I450 and N345/I336, located in the active sites of (−)-LS and (+)-LS, determine the binding direction of the substrate in the enzyme: when the binding direction of (−)-LS substrate is not correct, there will be a steric hindrance between the M458 residue and the substrate molecules, so that the substrate can only form the direction suitable for (−)-limonene synthesis. The I336 residue of (+)-LS also plays a similar role in the synthesis of (+)-limonene.

**Fig. 7:**
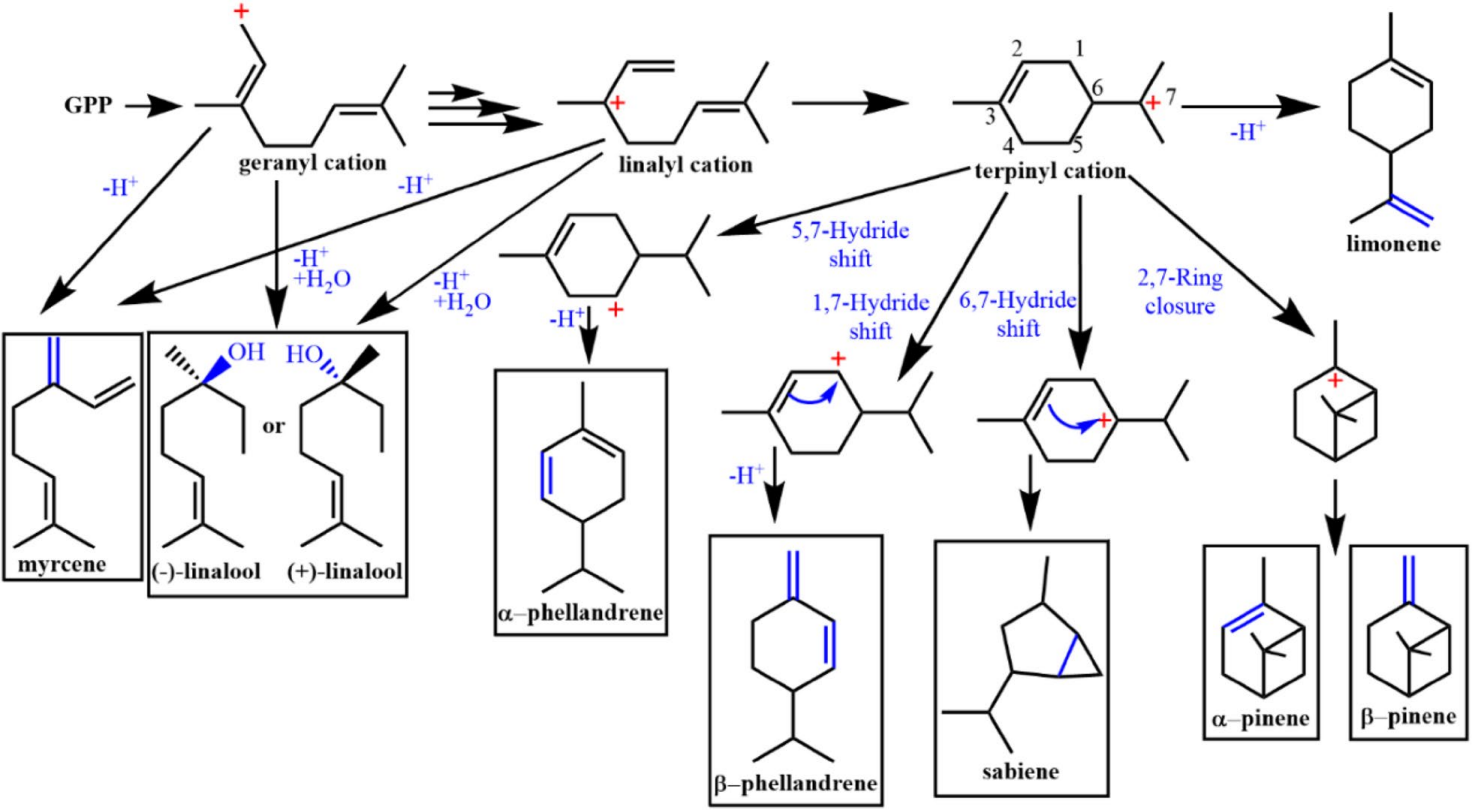
Proposed monoterpenes that can be achieved by limonene synthase variants. Linalyl cation and terpinyl cation are the common intermediate of monoterpene cyclases, which can produce many monoterpenes

Compared with other monoterpene cyclases, the LS-catalyzed cyclization is simpler: Limonene can be obtained by one-step deprotonation after configuration change, and the existence of intermediate LPP can be further verified by fluorinated substrate analogue 8,9-difluorogerany diphosphate (DFGPP) (Morehouse et al. 2019). If we have enough information of the structure–activity relationship between monoterpene cyclase and its substrate, limonene synthase might be used to catalyze the synthesis of more monoterpene skeleton molecules by mutating the key residues (Fig. 7) (Srividya et al. 2015; Xu et al. 2018). Xu et al. (2017) successfully converted (−)-LS into a pinene synthase (N345A/L423A/S454A) and a phellandrene synthase (N345I) by protein engineering of (−)-LS derived from *M. spicata*, thus revealing the plasticity of the active center of LS, providing a reference for redesign of monoterpenoid enzymes.

#### 1,8-Cineole synthase (CS)

1,8-Cineole, also known as eucalyptol, is the main component of eucalyptus oil, which has anti-inflammatory (Li et al. 2016), antiviral (Muller et al. 2016), anti-microbial and antioxidant (Seol and Kim 2016).

**Fig. 8:**
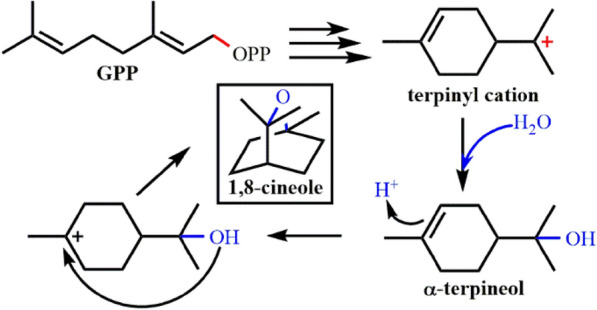
Cyclization reaction catalyzed by cineole synthase. Terpinyl cation is quenched by water molecule, producing 1,8-cineole through protonation induced cyclization reaction

In the process of CS-enzymatic reaction, α-terpinyl cation intermediates that commonly appear in the monoterpene cyclase-catalyzed reaction will also be formed (Croteau et al. 1994). Finally, carbocation will be eliminated by water molecules to form α-terpineol. This shows that solvent molecules have an important influence on the stability of reaction intermediates or the elimination of carbon positive ions in the catalytic reaction of terpene cyclases (Fig. 8).

The crystal structure of *Sf*CS from *Salvia fruticosa* (PDB ID: **2J5C**) showed it has αβ domains (Kampranis et al. 2007). Recently, as the first monoterpene cyclase from bacteria, the crystal structure of *Sc*CS from *Streptomyces clavuligerus* ATCC 27064 was analyzed (Karuppiah et al. 2017). It was found that *Sc*CS from bacteria contains only α domain, which is significantly different from Eucalyptus synthases from plants.

In *Sf*CS, N338 is a conserved residue in CS from *S. officinalis* and *Arabidopsis thaliana*, probably because its side chain can form hydrogen bonds with water molecule. Therefore, N338 may play a key role in controlling water molecules, eliminating carbocation intermediates and producing α-terpineol. After mutation, the variants did no longer produce cineole, instead they produced sabinene, limonene and a small amount of α/β-pinene and myrcene. This phenomenon further confirmed the important role of N338 (Kampranis et al. 2007). While in *Sc*CS, W58 and N305 regulate a water molecule, which may be crucial for the subsequent elimination of carbocation.

It is closely correlated to the structure of its substrate binding pocket why terpene cyclases can bind to a substrate with a specific chain length. The variant N338A of *Sf*CS expands the substrate pocket, which shifts its substrate from GPP (C10) to the sesquiterpene universal substrate FPP (C15), resulting in 49% *trans*-α-bergamotene (Kampranis et al. 2007). Karuppiah et al. (2017) compared the structure of *Sc*CS with a sesquiterpene cyclase, aristolochene synthase from *Aspergillus terreus* (*At*AS, PDB ID: **4KUX**), and a selinadiene synthase (SdS, PDB ID: **4OKZ**), and found that the two residues F77 and F179 limit the substrate combining pockets of *Sc*CS. Linalool/nerolidol synthase (*Sc*LinS, PDB ID: **5NX4**), from the same source as *Sc*CS, could produce linalool with GPP as a substrate, and nerolidol with FPP as a substrate. The amino acids of *Sc*CS at positions corresponding to F77 and F179 are T75 and C177, which are less sterically hindered. These two cases show that the substrate binding pocket of terpene cyclases has certain restrictions on the size of the substrate molecules, so that it can only accept substrates with suitable chain length entering the active center. The structural modification of the substrate pocket of the enzyme may produce more new cyclase molecules that may accommodate different chain lengths.

### Sesquiterpene cyclases

#### α-Bisabolol synthase (α-BOS)

α-Bisabolol is a sesquiterpenoid alcohol with antibacterial and anti-inflammatory effects. α-Bisabolol and its derivatives are potential cancer treatment drugs which have anti-cancer activity through inhibiting the serine/threonine kinase (Brehm-Stecher and Johnson 2003; Kim et al. 2011; Murata et al. 2017; Seki et al. 2011).

The terpene cyclases corresponding to the 4 isomers of α-Bisabolol have been discovered (Albertti et al., 2018; Attia et al. 2012; Muangphrom et al. 2019; Nakano et al. 2011). Li et al. (2013) obtained the crystal structure (PDB ID: **4FJQ**) of an α-bisabolol synthase (*Aa*BOS) from *Artemisia annua*. *Aa*BOS has αβ domains and the active center is located in the α domain. The DDXXD characteristic sequence is located in the D helix, and the (N,D)DXX(S,T)XXXE metal binding motif is located in the H helix, except G replaces S/T in *Aa*BOS. Previous studies have shown that G can retain the ability of S/T to bind metal ions in plant terpene cyclase (Zhou and Peters 2009).

**Fig. 9:**
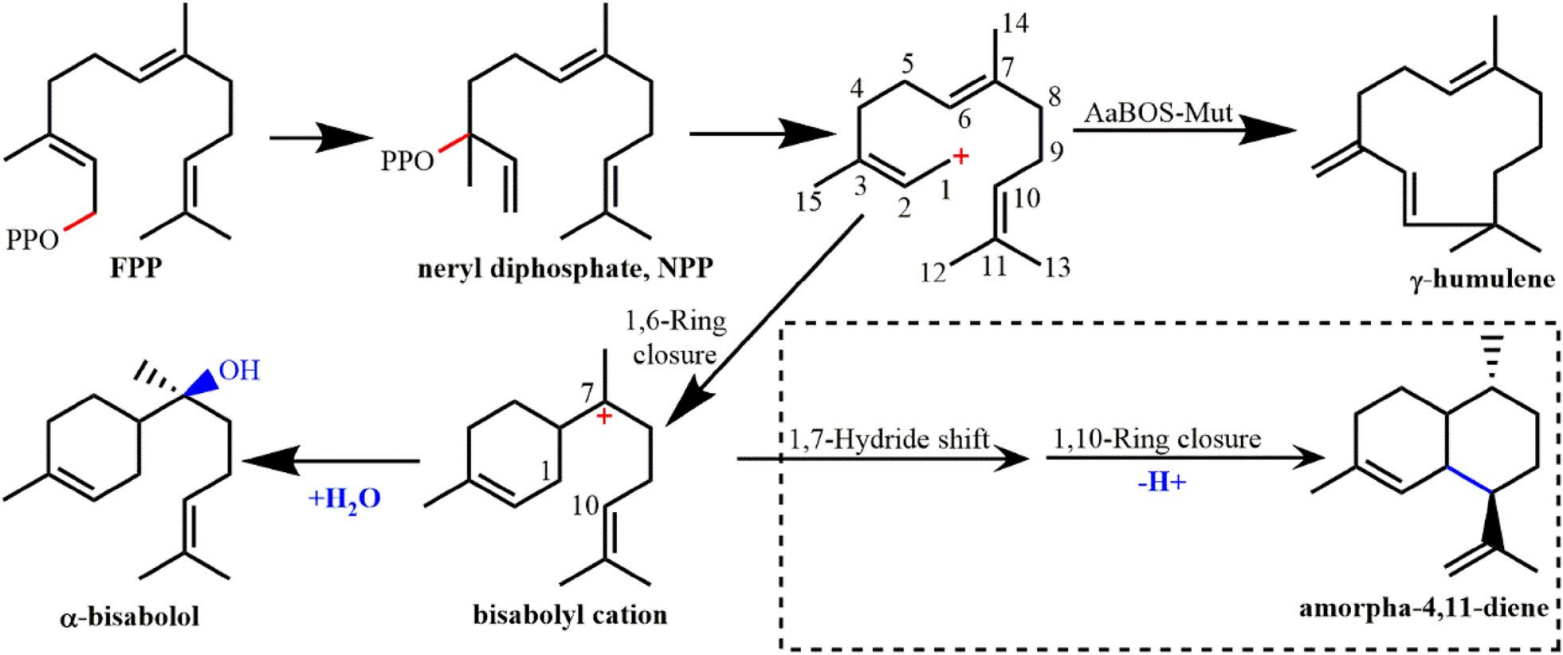
Cyclization reaction catalyzed by α-bisabolol synthase from *Artemisia annua* (*Aa*BOS). Dashed box shows the reaction catalyzed by amorphadiene synthase. *Aa*BOS-Mut represents the *Aa*BOS mutant (V373N/L381A/I395V/N398I/L399T) (Li et al. 2013)

*Aa*BOS catalyzes the cyclization reaction of FPP (Fig. 9), and the carbocation intermediate is finally eliminated by water molecules to produce the hydroxylated product α-bisabolol (Li et al. 2013). Based on the crystal structure, a five-site mutant *Aa*BOS-Mut (V373N/L381A/I395V/N398I/L399T) that could produce γ-humylene as the major product (68.8%) was identified (Li et al. 2013). The structure–activity relationship of the mutant was investigated, indicating that the mutation L399T is essential for the production of γ-humylene.

Amorphadiene synthase (*Aa*ADS) from *A. annua* was found to catalyze the first step of artemisinin biosynthesis, which has an 82% identity with the *Aa*BOS sequence. The study on structural mechanism of *Aa*BOS provides a reference for the related researches of *Aa*ADS (Li et al. 2013). As the crystal structure of amorpha-4,11-dime synthase is not available, *Aa*BOS can be used as an excellent template for homologous modeling of *Aa*ADS. Recently, the (+)-α-bisabolol from *Artemisia kurramensis* and *Artemisia maritima* has been found. These two BOSs produced the unique product of (+)-α-bisabolol, revealing their high enantioselectivity and product specificity. Enantiomeric pure (+)-α-bisabololl can be de novo synthesized in yeast expression system, in which the yield was about 83 mg/L (Muangphrom et al. 2016).

The regulatory mechanism of terpenes on water molecules is currently unclear. The final catalytic step of α-bisabolene synthase is to eliminate carbocations through water molecules, while α-bisabolene synthase gets non-hydroxylated products through deprotonation. The crystal structure of α-bisabolene synthase (*Ag*BOeS) derived from *Abies grandis* has been obtained (McAndrew et al. 2011), which has a αβγ three-domain structure, with *k*_cat_/*K*_M_ = 38 M^−1^ s^−1^. If the eutectic structure of *Aa*BOS and *Ag*BOeS was obtained using alternative substrates, the regulation mechanism of *Aa*BOS on water molecules may be obtained by structural analysis and comparison, which may provide reference for the study of the regulation mechanism of terpene cyclases on water molecules. Bisabolane, a saturated alkane of α-myxol, is a kind of biofuel (Peralta-Yahya et al. 2011). It can be synthesized by α-myxol or α-myrcene, but the cost of biological synthesis is too high. If the catalytic efficiency of cyclase can be improved by protein engineering, the cost will be reduced. For this reason, the research on the interaction and structure–activity relationship between enzyme and substrate is very important.

#### *epi*-Isozizaene synthase (EIZS)

**Fig. 10:**
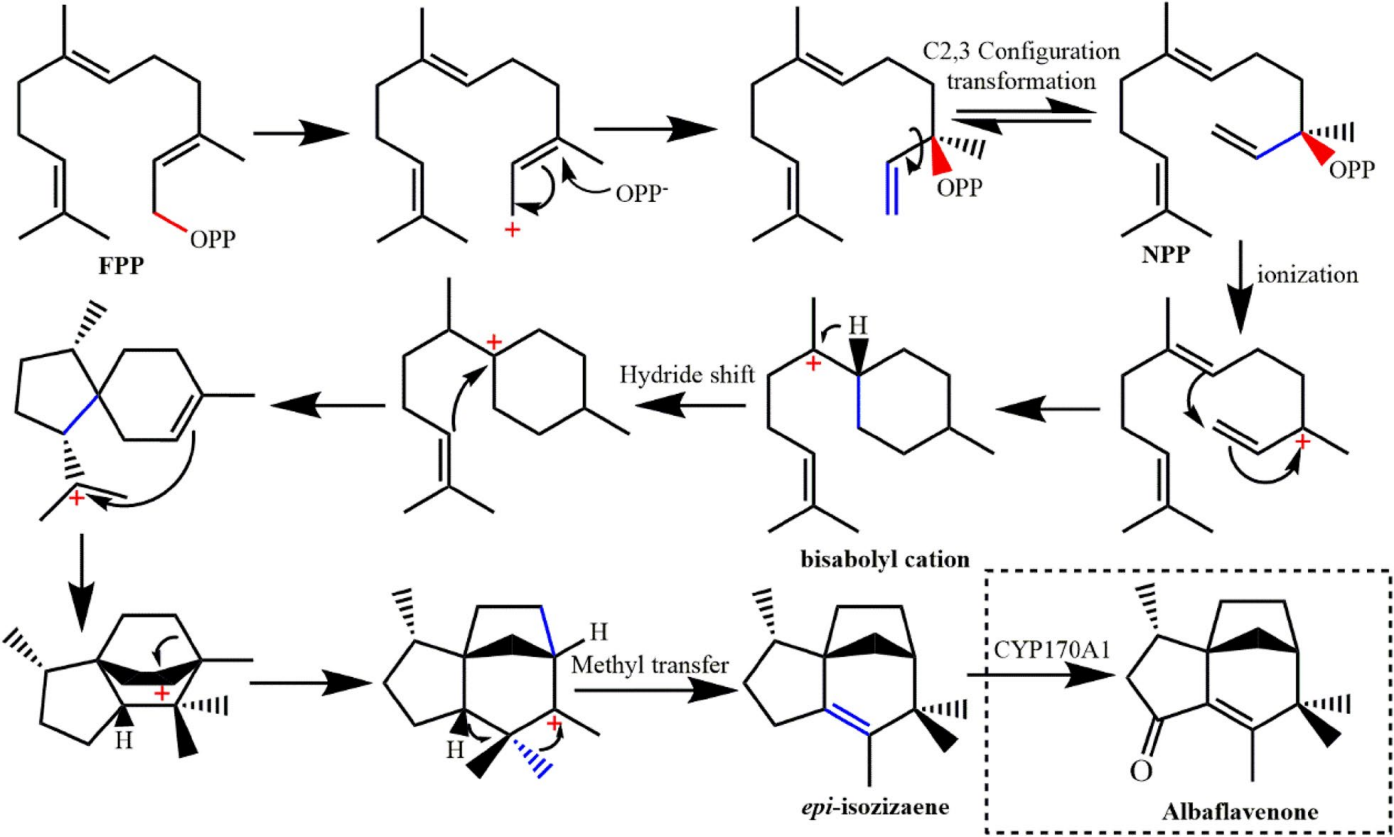
Cyclization reaction catalyzed by EIZS. Dashed box shows the formation of albaflavenone

Albaflavenone is synthesized by cyclization of FPP catalyzed by enzyme EIZS followed by two-step continuous oxidation of CYP170A1 (Fig. 10) (Zhao et al. 2008). The EIZS-catalyzed cyclization, goes through the steps of hydrogen transfer and methyl transfer after the formation of bisabolyl cation, finally forming tricyclic sesquiterpenes. The precise regulation of the above transfer processes shows the important role of enzymes.

**Fig. 11:**
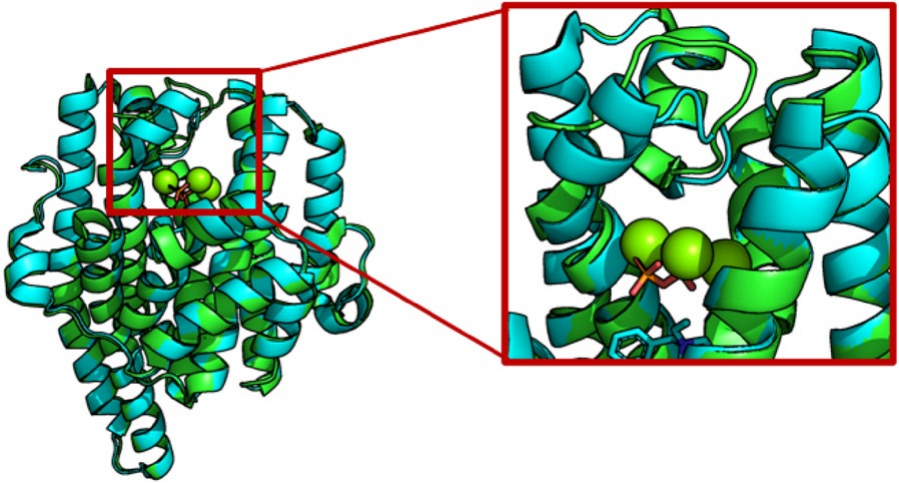
Superposition of unliganded D99N *epi*-isozizaene synthase (PDB ID: **3LGK**, green) and *epi*-isozizaene synthase complex with Mg, inorganic pyrophosphate and benzyl triethyl ammonium cation (PDB ID: **3KB9**, cyan). The red box shows the details of conformational difference

The crystal structure of EIZS from *Streptomyces coelicolor* A3 (2) (Fig. 11) has been solved (PDB ID: **3KB9**), indicating that EIZS has only an α domain (Aaron et al. 2010). While being located by Mg^2+^, the bound pyrophosphate group also forms hydrogen bonds with R194, K247, R338 and Y339. These interactions stabilize the closed conformation of EIZS and promote the ionization of pyrophosphate group. By comparing the crystal structure of EIZS D99N without binding ligand and that of the EIZS complex with pyrophosphate group, it is found that the helix and loop of the active center have obvious conformational changes, making the solvent molecules discharge from the active pocket and preventing the premature elimination of carbonium ion intermediates. Using benzyltriethylammonium cation (BTAC) to simulate the reaction intermediate bisabolyl cation, it was found that the cation intermediate is stabilized by the electrostatic action of pyrophosphate anion and the cation–π interactions of F95, F96, and F198. Mutation of these aromatic amino acid residues will break such stabilizing effects and may cause the premature elimination of carbocations, thereby producing other cyclized molecules.

**Fig. 12:**
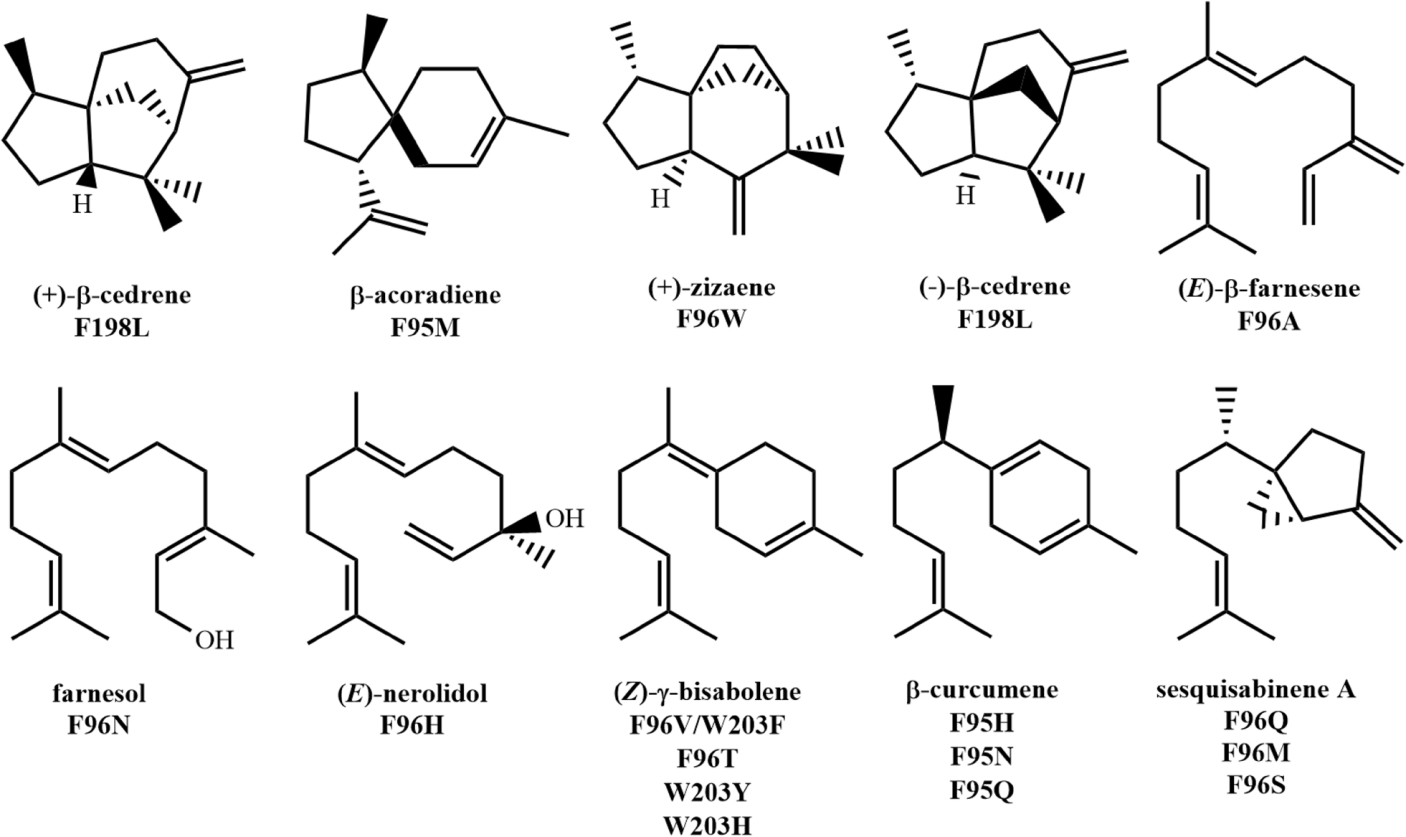
Predominant cyclization products of EIZS mutants. Main products of the corresponding mutants were summarized from refs. (Aaron et al. 2010; Blank et al. 2017; Li et al. 2014)

The contour of the terpene cyclase activity pocket is very similar to the product structure. When this contour does not match the product molecule well, more by-products will be produced. The EIZS-catalyzed reaction produces 80% of epi-isozizaene and minority of other sesquiterpene products, indicating a certain degree of promiscuity of the cyclase. By replacing some hydrophobic residues with polar amino acids, the contour of the active pocket can be changed (Blank et al. 2017; Li et al. 2014), allowing the enzyme to produce more valuable cyclized molecules (Fig. 12). The product of variant F95H (Li et al. 2014) was β-curcemene (50% purity), which can be transferred into biofuel bisabolene after hydrogenation. The measured *k*_cat_/*K*_M_ of this variant was 2600 M^−1^ s^−1^, which is 70 times higher than that of α-bisabolene synthase mentioned before “α-Bisabolol synthase (α-BOS)” section), indicating the high catalytic efficiency of this mutant enzyme. By analyzing the crystal structure of the F95Q mutant (PDB ID: **6OFV**), it was found that the active pocket of the F95Q mutant is enlarged, which makes the binding of FPP more flexible, thereby affecting the precise control of the original enzyme on the cyclization reaction (Blank et al. 2019). The polarity change of this site also lets new water molecules enter the active pocket. In the crystal structure, the conformation of the benzyltriethylammonium cation, which mimics the reaction intermediate bisabolyl cation, is changed by the influence of water molecules.

#### Aristolochene synthase (AS)

The bicyclic sesquiterpene aristolochne is the precursor material for the synthesis of gigantenone, sporogen-AO1, bipolaroxin, PR-toxin and other mycotoxins (Cane and Kang 2000). The crystal structures of aristolochne synthases derived from *Penicillium roqueforti* (*Pr*AS, PDB ID: **1DI1**) (Caruthers et al. 2000) and *A. terreus* (*At*AS, PDB ID: **2OA6**) (E.Y. Shishova et al. 2006) have been resolved. The identity of the two protein sequences is 61%. The amino acids in the active site are highly conserved, with different residues located mainly on the protein surface.

*Pr*AS is a class I terpene cyclase with a single alpha domain (Caruthers et al. 2000). The isoprene chain of the substrate will start the reaction on the hydrophobic interface composed of F112, F178, L108, L111 and G205. While the residues of R200, K251, Y341 and R340 can interact with pyrophosphate groups, metal ions and DDXXD/E motifs through hydrogen bonds or salt bridges to stabilize the binding of pyrophosphate groups, which is very important for the conformational transformation of enzymes (Faraldos et al. 2012). This stabilization effect is common in terpene cyclases, such as BPPS (as mentioned in “Bornyl diphosphate synthase (BPPS)” section) and EIZS (as mentioned in “e*pi*-Isozizaene synthase (EIZS)” section).

The template role of the enzyme in the entire cyclization reaction is very important. Through site-directed mutagenesis, the roles of multiple residues were discovered: Y92 controls FPP to facilitate the conformation of cyclization and folds it into (*S*)-(−)-germacrene A (Calvert et al. 2002); W334 and F112 can stabilize the carbocation intermediates by cation–π interaction (Faraldos et al. 2011a; Forcat and Allemann 2006); F178 promotes the formation of FPP cyclization and eudesmane cation, and F178 also interacts with F112 to co-stabilize the transition state of the cyclization on the reaction (Forcat and Allemann 2006), while L108, Y92 and F112 jointly control FPP to form the correct conformation (Faraldos et al. 2011b). *Pr*AS can also convert the unnatural substrate 7-methylene farnesyl diphosphate to aristolochne and a small amount of valenene (Faraldos et al. 2016), showing that *Pr*AS has the potential for protein engineering and mutation of key amino acids in the catalytic process to get access to a variety of products (Calvert et al. 2002; Faraldos et al. 2011b; Felicetti and Cane 2004; Forcat and Allemann 2006).

*At*AS is also a class I terpene cyclase with a single α domain (Shishova et al. 2006), and its residues R175, K226, R314 and Y315 have the same effect as the corresponding sequence of *Pr*AS. The *At*AS crystals have a tetramer structure. However, the presence of dimer enzymes was verified through gel analysis. Previous studies have found that the activity of *At*AS decreased when its concentration was higher than 27 nM (Felicetti and Cane 2004). After further research, it was inferred that the tetramer structure may inhibit the enzyme activity. Chen et al. (2013) obtained the eutectic structure of *At*AS with substrates and carbocation intermediate analogs, making the study of the structure–function relationship between the enzyme and the substrate more insightful. Van der Kamp et al. (2013) proposed the dynamic process of *At*AS conformational change through molecular dynamics (MD) simulation: at first the substrate molecule binds followed by the binding of two Mg^2+^ cations, the enzyme conformation changes from open to closed state, and then the last Mg^2+^ ion combines and starts the cyclization reaction. When the reaction is finished, the product molecule and two Mg^2+^ ions are released first, the pyrophosphate and Mg^2+^ are released finally. There are water molecules at the active site of *At*AS in the reaction state (Chen et al. 2016a), but the final product is not a hydroxylated molecule, indicating that these water molecules have relatively fixed binding positions by the hydrogen bonding of surrounding residues. They do not directly participate in the cyclization reaction but participate in the network of molecular conformation control.

Zhang et al. (2016a, b) studied *At*AS, farnesyl diphosphate synthase (FPPS) and 5-*epi*-aristolochene synthase (EAS) using QM(DFT)/MM and MD simulation, concluding that the protonation state of the pyrophosphate group is different among various enzymes, which is closely related to the enzyme structure and its microenvironment. In *At*AS, it was presumed to be in a deprotonated state (PP_i_^3−^); in FPPS, it was speculated to be in a single-protonated state (PP_i_^2−^); EAS was in a double-protonated state (PP_i_^−^). This finding allows a better understanding of the mechanism on cleavagee of pyrophosphate groups.

#### 5-epi-Aristolochene synthase (or epi-aristolochene synthase, EAS)

Capsidiol is a phytoalexin that can resist fungal or bacterial infections. It is catalyzed from FPP by EAS and 5-*epi*-aristolochene hydroxylase (EAH) (Li et al. 2015). EAS from *Nicotiana tabacum* is the first plant terpene cyclase obtained a crystal structure. The structure shows that the enzyme is a monomer containing αβ domain, including the class I terpene cyclase DDXXD and (N,D)DXX(S,T)XXXE metal binding motif. Its active center was determined through the crystal structure analysis of the complex of FPP analog and enzyme (Starks et al. 1997).

R264 of EAS directly interacts with phosphate group, which keeps the position of the substrate. R266 interacts with Y527 and T528 to make the enzyme semi closed, and then R266 reverses its conformation to form a salt bridge with E531, thus making the conformation completely closed (Gennadios et al. 2009; Rising et al. 2015; Zhang et al. 2016a). The N-terminal β domain stabilizes the helix and loops of the active site, without which the R266–E531 salt bridge will be broken, resulting in the failure of forming closed conformation. W273 and Y527 of the active pocket play an important role in the localization and stabilization of the substrate. When the substrate and Mg^2+^ enter the substrate pocket, the W273 generates π–π stacking effect with the C_6,7_ double bond of substrate, making the substrate more reactive and promoting the C_1,10_ ring-closure process. Y527 stabilizes the reaction intermediate through cation–π interaction, and also helps the conformational transformation of the enzyme. If Y527 is mutated, it will make the active site more flexible, and it is unable to form a closed conformation (Zhang et al. 2016a).

Compared with the AS-catalyzed reaction, EAS has a different product configuration, which shows that the active pocket of terpene cyclase is an important template for substrate folding. Y520 of EAS acts as a proton donor to catalyze the protonation of the intermediate (*R*)-(+)-germacrene A (Starks et al. 1997). It is inferred that Y92 in *Pr*AS has the same function, but Y92 of *Pr*AS does not align with Y520 of EAS, which may be more conducive to form products with different configurations (Caruthers et al. 2000). O'Brien et al. (2016) combined quantum mechanics with computational docking to construct an all-atom model of all possible reaction intermediates in the active pocket of EAS, which has great significance for structure analysis of enzyme and protein engineering.

Zhang et al. (2019) studied the fidelity of *At*AS and the promiscuousness of EAS using QM/MM and found that the D444–Y520 dyad acts as a pair of additional acid–base residue pairs to increase the promiscuousness of EAS. In *At*AS, F81/F147 stabilizes the carbocation intermediate through steric hindrance and cation–π interaction, which enhances the fidelity of *At*AS.

The identity of *N. tabacum* 5-*epi*-aristolochene synthase and *Hyoscyamus muticus* premnaspirodiene synthase is 75%. O'Maille et al. (2008) found that there are 9 key residues that may affect the final product of these enzymes. EAS was successfully transformed into premnaspirodiene synthase by replacing amino acid residues at the 9 positions. By comparing structures of *N. tabacum* 5-*epi*-aristolochene synthase, *H. muticus* premnaspirodiene synthase with their mutants (Koo et al. 2016), it is found that the catalytic promiscuity of terpene cyclase is related to the stability of the enzyme itself: the increase of stability will reduce the number of conversions and decrease the diversity of by-products. This is because the increased rigidity of the enzyme reduces the flexibility of conformational changes, resulting in slow product release, thereby increasing the enzyme’s template effect on the cyclization reaction and reducing the generation of by-products. These studies deepen our understanding on the catalytic promiscuity of terpene cyclases, and provide new ideas for protein engineering of these terpene cyclases. EAS has the ability to catalyze unnatural substrates, and it can simultaneously utilize unnatural substrates (*cis*, or *trans*)-FPP and natural substrates (*trans*,* trans*)-FPP to produce *epi*-aristolochne (Zhang et al. 2018). EAS can also catalyze the synthesis of anilinogeranyl diphosphate to form a new macrocyclic alkaloid 3,7-dimethyl-*trans*, *trans*-3,7-azaparacyclophane-diene (geraniline) (Rising et al. 2015). These studies have shown the flexibility of proteins and the possibility that those enzymes might act as good start points for extensive molecular evolution.

### Diterpene cyclases

#### Taxadiene synthase (TS)

Paclitaxel is a class of tetracyclic diterpenoids containing multiple oxygen functional groups. It is the first tubulin stabilizer that can make cells stay in G2–M stage by promoting tubulin aggregation and stabilizing microtubules, leading to cell apoptosis and achieving the purpose of anti-cancer (Yang and Horwitz 2017). In 1992, paclitaxel was approved by the U.S. Food and Drug Administration (FDA) for the treatment of ovarian cancer, and was subsequently used in the treatment of breast cancer, head cancer, lung cancer and other cancers, becoming one of the most popular anti-cancer drugs in the world (Liu et al. 2016).

**Fig. 13:**
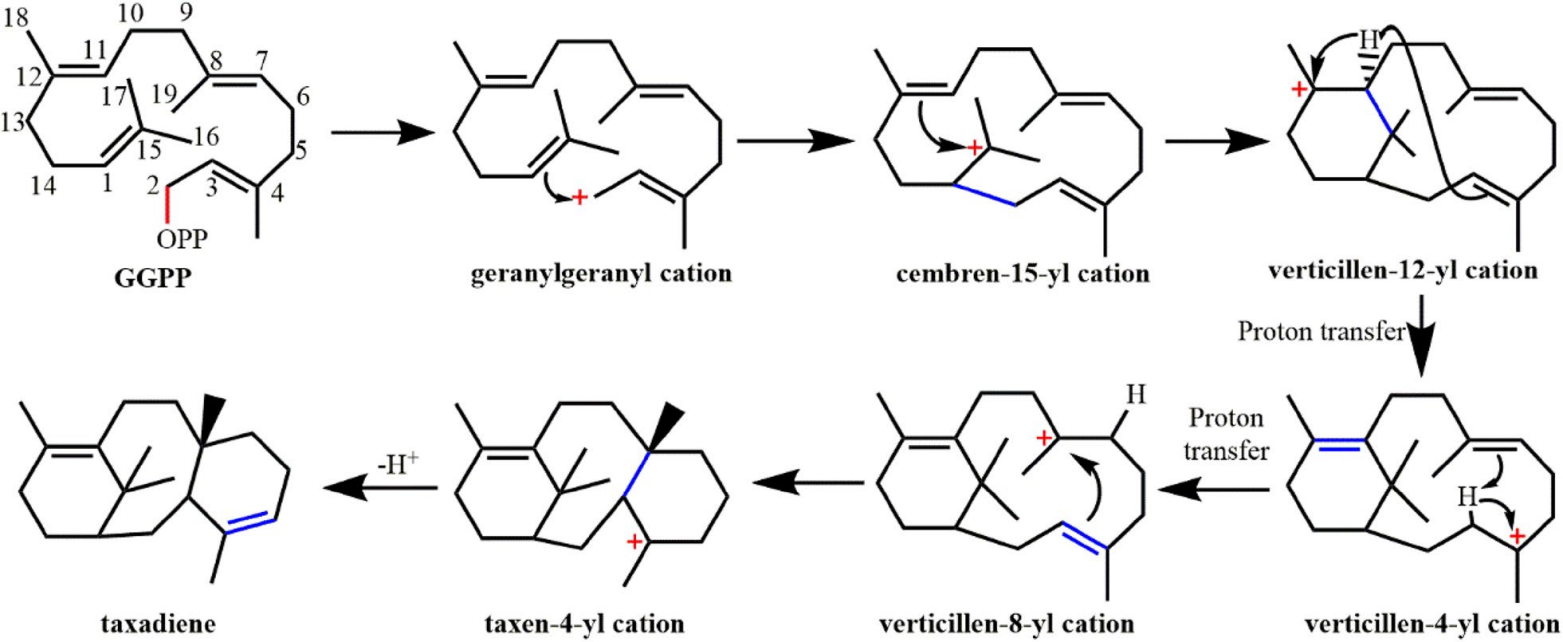
Formation of taxadiene from geranylgeranyl pyrophosphate (GGPP) by taxadiene synthase

Taxadiene, the skeleton structure of paclitaxel, is generated by GGPP cyclization (Fig. 13). The reaction includes the ionization activation of pyrophosphate groups, proton transfer, deprotonation and elimination of carbocations (Koksal et al. 2011; Lin and Hezari 1996; Schrepfer et al. 2016).

**Fig. 14:**
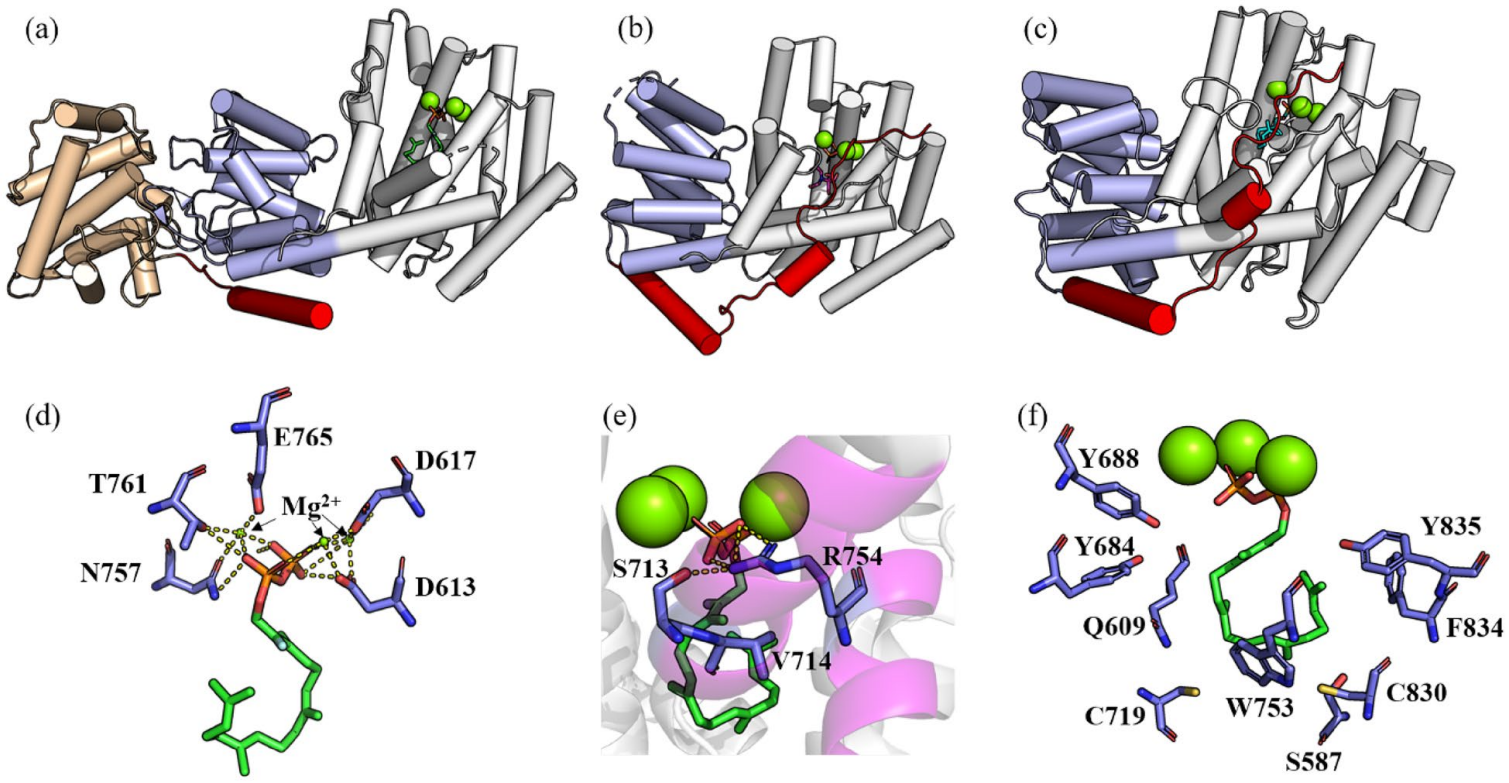
**a** Structure of TS complexed with 2-fluorogeranylgeranyl diphosphate and Mg^2+^ (PDB ID: 3P5R). **b** Structure of BPPS complexed with inorganic pyrophosphate and Mg^2+^ (PDB ID: 1N1Z). **c** Structure of EAS complexed with substrate analog farnesyl hydroxyphosphonate and Mg^2+^ (PDB ID: 5EAT). **d** Metal-binding motifs of taxadiene synthase. **e** Effector triad of taxadiene synthase which sites in the helix that is shown in magenta. **f** Key aromatic and polar residues of taxadiene synthase. Mg^2+^ is in green; α, β, & γ domains are in white, light blue and wheat, respectively; The red shows N-terminal segment which ‘caps’ the active site

Koksal et al. (2011) got the crystal structure of TS from *T. brevifolia*, complexed with Mg^2+^, 13-aza-13,14-dihydrocopalyl diphosphate (ACP) or 2-fluorogeranylgeranyl diphosphate (FGP) (TS-ACP, PDB ID: **3P5P**; TS-FGP, PDB ID: **3P5R**). This is the first crystal structure of diterpene cyclases, which contains the αβγ domain (Fig. 14a). The catalytic site is located in the α domain, and contains the typical class I terpene cyclase metal binding motifs, D^613^DMAD and N^757^DTKTYQAE. But this crystal structure is inactive with the transport sequence cut and an additional 27 amino acid residues truncated.

Just like the N-terminal of BPPS (Whittington et al. 2002) and EAS (Starks et al. 1997) (Fig. 14b, c), the truncated N-terminal sequence is above the active site and can cover the active site, transforming the enzyme into a closed conformation which is necessary for the cyclization reaction. However, because the crystal structure obtained so far does not contain this N-terminal sequence; therefore, it only represents the structure of an inactive polypeptide, instead of the native enzyme. The conformation is not in the closed state required for the catalytic reaction, but presents a half-closed state (Koksal et al. 2011), which makes it difficult to study the structure–function relationship between enzyme and substrate. Therefore, the structural mechanism of taxadiene synthase is mostly elucidated based on homology modeling, quantum mechanical simulation and molecular dynamics calculations.

Schrepfer et al. (2016) employed the crystal structures of BPPS and its complex with Mg^2+^ and 3-azageranyl diphosphate (PDB ID: **1N20**) (Whittington et al. 2002) as templates, constructed a TS structure model in the closed conformation. Combining with energy minimization and MD simulation, they analyzed the conformational changes and key residues involved in the entire cyclization reaction of TS. The results of the simulation analysis were verified by site-directed mutagenesis. Through this model, it is found that the effector triad of taxadiene synthase (i.e., the pyrophosphate group receptor R754, the linker S713, and the effector V714-O) are closely related to the ionization of the pyrophosphate group. The receptor R754 will deflect first to form a hydrogen bond with the pyrophosphate group of substrate, while the linker S713 also deflects and forms a hydrogen bond with V714-O. This conformational change causes the carbonyl oxygen (G182-O) of the effector G182 to flip into the active center, which is important to the formation of the closed conformation and the ionization of the pyrophosphate group. This effector triplet is widely present in class I terpene cyclase, but the spatial position of the triplet is different among different sources (Baer et al. 2014). Based on the model obtained in former research, Van Rijn et al. (2019) further explored the cyclization mechanism of taxadiene synthase using the QM/MM method and found that due to the influence of the position, orientation and conformation of the cation, as well as the electrostatic interaction between the active site structure and the influence of water molecules, the carbocation rearrangement catalyzed by TS has significant sensitivity to the change of intramolecular microenvironment. Freud et al. (2017) figured out the proton transfer step of the TS enzymatic cyclization process using the QM/MM method. From the energy point of view, this work revealed the path in which verticillen-12-yl cation is transferred directly, giving verticillen-8-yl cation in one step, which is more advantageous than the indirect process going through verticillen-4-yl cation.

The aromatic group of the protein is essential to stabilize the carbocation intermediate during the cyclization reaction. For example, W753, F834, Y835, and Y841 can stabilize carbocations through cation–π interactions. By mutating these residues, the cyclization cascade can be terminated early to obtain the corresponding cyclization products, which is of great significance to the study of mechanism and the design of synthetic methods for terpenoid cyclization. Mutants W753H and Y841F can produce the main products, cembrene A and verticillia-3,7,12(13)-triene, respectively (Schrepfer et al. 2016). Ansbacher et al. (2018) constructed a structural model of the W753H mutant, revealing energetically that the mutant is more conducive to deprotonation of the intermediate to form cembrene A. Polar residues S587, Q609, Y684, Y688, C719 and C830 may act as catalytic bases for the deprotonation of taxadiene (Koksal et al. 2011). Edgar et al. (2017) examined the effects of these residues and found that the mutant Q609G gives verticillia-3,7,12(13)-triene as the unique product, while the mutant Y688L can produce taxa-4(20),11(12)-diene which is more conducive to the hydroxyl of taxadiene-5α-hydroxylase (CYP725A4) (Jennewein et al. 2004), thus initiating the first step of converting taxene into the anticancer molecule, Paclitaxel.

Pemberton et al. (2017) investigated the function of βγ domains of TS and found that when the βγ domains are removed, TS loses its cyclization ability; when only removing the γ-domain, the catalytic activity will be greatly reduced; the β-domain also has some influences on the fidelity of the enzyme, indicating that there exists a certain interaction between the functional domains of the enzyme.

#### Cyclooctat-9-en-7-ol synthase (CotB2)

**Fig. 15:**
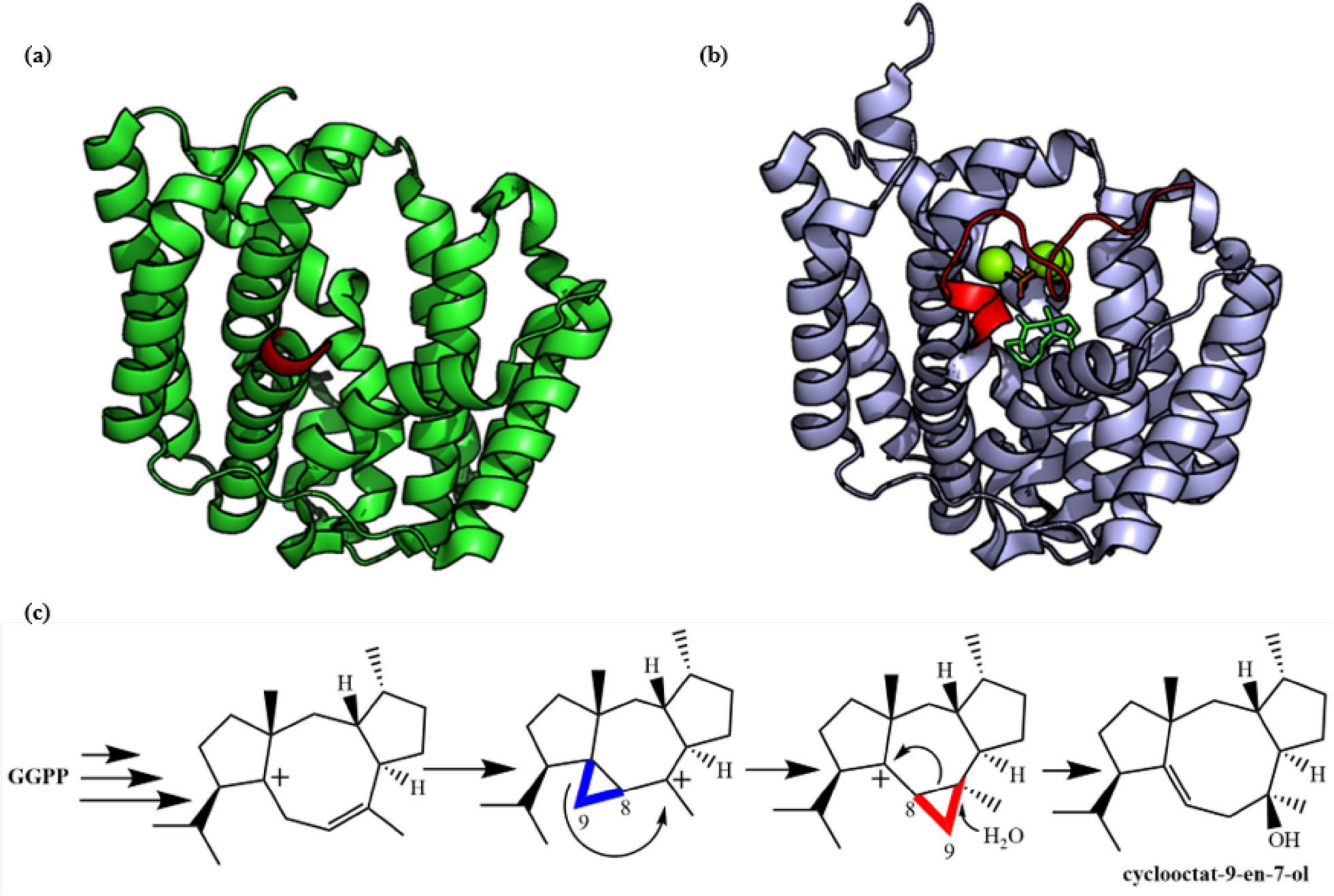
Structure and function of CotB2. **a** Crystal structure of the bacterial diterpene cyclase CotB2 (PDB ID: **4OMG**). C-Terminal is in red. **b** Crystal structure of CotB2 in complex with 2-fluoro-3,7,18-dolabellatriene (PDB ID: **6GGI**). C-Terminal is in red. **c** Reaction catalyzed by CotB2

Janke et al. (2014) obtained the crystal structure of CotB2 from *Streptomyces melanosporofaciens*, which is the first crystal structure of diterpene cyclase from bacteria (PDB ID: **4OMG**), but this protein is an inactive enzyme with 15 N-terminal residues and 10 C-terminal residues truncated (Fig. 15a). In the following research, Driller et al. (2018) successfully obtained the eutectic structure (PDB ID: **6GGI**) of CotB2 with Mg^2+^ and a substrate analogue, 2-fluoro-3,7,18-dolabellatriene. This structure presents a closed conformation in the reaction state, and the peptide chains which were cut off at the C-terminal before blocking the active center of the enzyme, such as a lid (Fig. 15b).

CotB2 belongs to class I terpene cyclase, but the sequence of its metal binding module DDXD is different from the typical DDXXD sequence: a kink is introduced near the proline residue of the third aspartic acid in this motif, which makes the α-helix D containing rich aspartic acid motif is shorter than other class I terpene cyclases (Janke et al. 2014). CotB2 also has effector triad, receptor R177, linker D180 and effector G182-O, but the triplet structure has less influence on the enzyme conformation (Tomita et al. 2017). This may be due to the fact that R177 and D180 are already connected through salt bridges in the open conformation, which reduces the conformational changes after substrate binding (Driller et al. 2019).

**Fig. 16:**
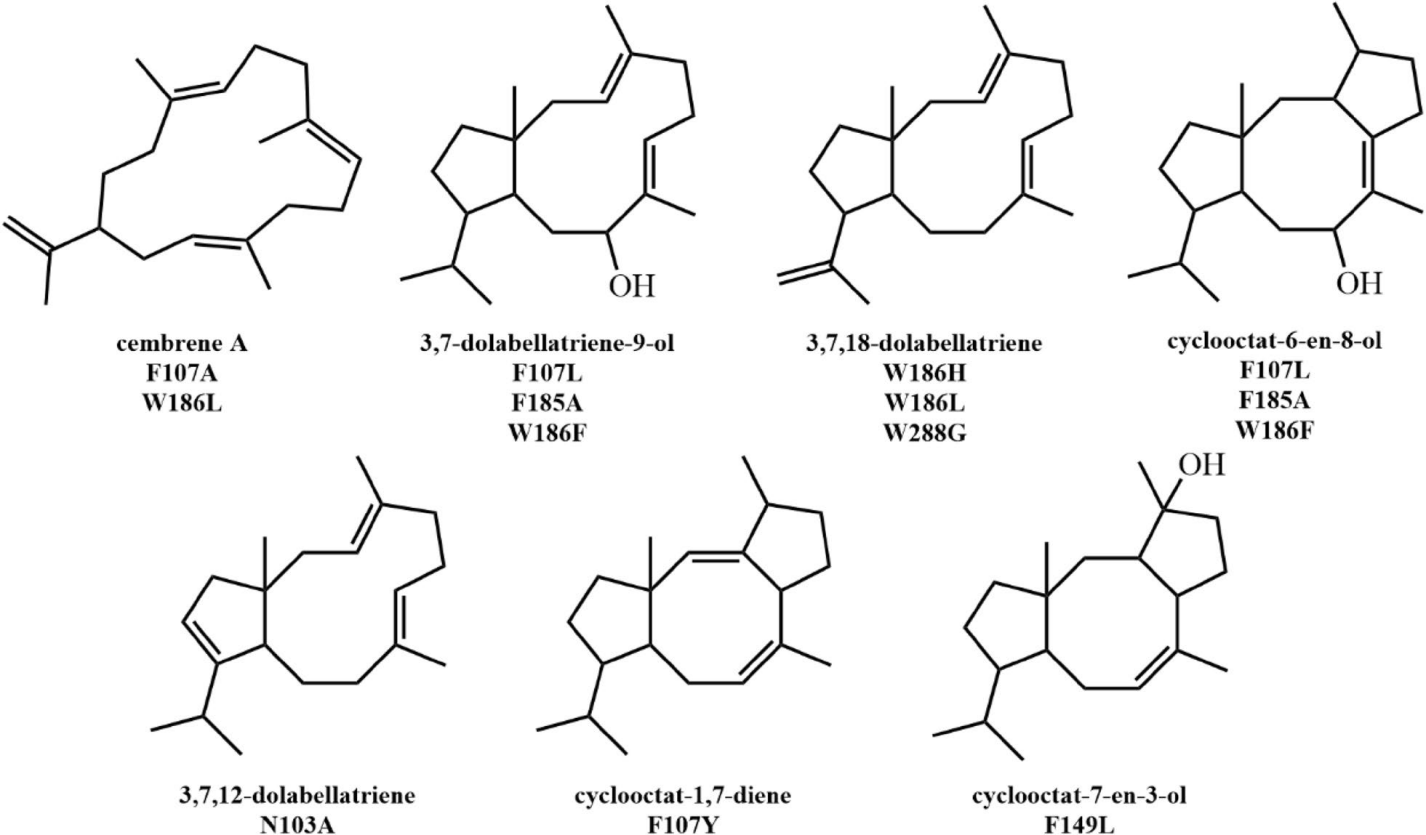
Products of CotB2 mutants. Products of the corresponding mutants were summarized from refs. (Görner et al. 2013; Janke et al. 2014; Tomita et al. 2017)

CotB2-catalyzed reaction involves special carbon rearrangement phenomenon (Meguro et al. 2015). C_8,9_ will rearrange during the cyclization (Fig. 15c), which shows the special synthetic ability and precise stereochemical control of the terpene cyclase. The hydroxylation products of CotB2 were obtained by eliminating carbon positive ions with water molecules. In the reaction, the active pocket of terpene cyclase contains some water molecules, which can stabilize the intermediates by forming hydrogen bonds. At the same time, water molecules have the ability to eliminate carbon positive ions, which are affected by the hydrogen bonds of surrounding amino acids without eliminating carbon cations. Site directed mutagenesis of these residues may result in inactivation of the enzyme, or more product molecules may be produced (Fig. 16) (Görner et al. 2013; Janke et al. 2014; Tomita et al. 2017). Assisted by some computational technologies, the reaction mechanism of CotB2 and its mutants are well studied that pointing the way for future terpene cyclase design (Raz et al. 2020; Tang et al. 2020).

### Sesterterpene cyclase

#### Sesterterpene cyclase from *Streptomyces mobaraensis* (SmTS1) with multiple products

**Fig. 17:**
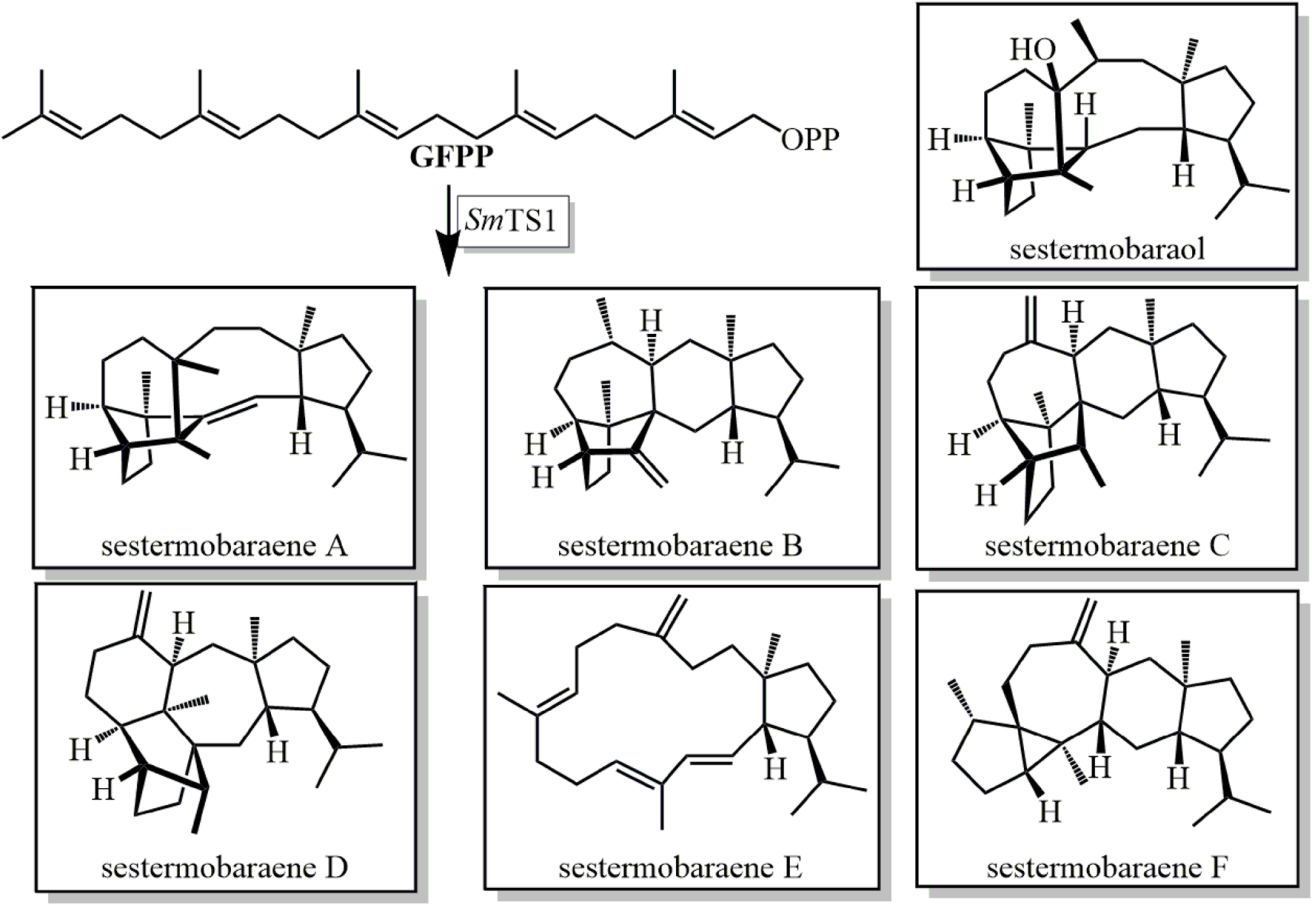
Products of *Sm*TS1

Recently, Hou and Dickschat (2020) reported a geranylfarnesyl diphosphate synthase (*Sm*GFPPS), which is the first bacterial geranylfarnesyl diphosphate (GFPP) synthase (GFPPS) and a multiproduct sesterterpene synthase (StTS) from *Streptomyces mobaraensis* (*Sm*TS1). SmTS1 can convert GFPP into a mixture of sesterterpene hydrocarbons and one sesterterpene alcohol (Fig. 17). The reaction is initiated by protonation of GFPP. Isotope labeling experiment revealed that different modes of hydrogen and methyl migrations resulted in different products and the quenching of cations by water molecules causes the sesterterpene alcohol. Starting from the ophiobolin F synthase from *Aspergillus clavatus*, a few StTSs from fungi (Chiba et al. 2013; Matsuda et al. 2016, 2015; Mitsuhashi et al. 2017; Okada et al. 2016; Qin et al. 2016; Ye et al. 2015) and plants (Huang et al. 2018, 2017; Shao et al. 2017) were discovered recently. In plants clustered genes for two discrete GFPPS and sesterterpene synthase are found, while in fungi sesterterpene biosynthesis is always promoted by bifunctional enzymes containing a C-terminal trans-prenyltransferase (PT) and an N-terminal TPSs domain. The PT domain produces the universal C25 sesterterpene precursor GFPP which is then cyclized by the TPS domain to form diverse scaffolds.

Although most StTSs produce various sesterpene backbones via class-I cyclization mechanism, AtTPS06 from *A. thaliana* utilizes a class-II cyclization mechanism (Chen et al. 2020b). Sesterterpennoids are amongst the rarest of all isoprenoids with approximately 1300 compounds known, which are widely distributed in terrestrial fungi, cyanobacteria, lichens, higher plants, insects, and various marine organisms and exhibit noteworthy biological activities (Li and Gustafson 2020; Liu et al. 2007; Wang et al. 2013). Apart from the remarkable biological activities of sesterterpenoids, complex molecular structures of sesterpenoids attract many chemist and biologist to explore great potential of sesterpenoids (Guo et al. 2021).

## Class II terpene cyclases

Class II terpenoid cyclases initiate catalysis by protonation of a carbon–carbon π bond or epoxide moiety in an isoprenoid substrate to yield a carbocation. These enzymes contain the characteristic sequence motif DXDD which is unrelated to the aspartate-rich motif of class I terpene synthases. The β and γ domains compose the active site of class II terpene synthases. Here, we introduce several class II terpene synthases to enhance the understanding of enzyme structure and function.

### ent-Copalyl diphosphate synthase (ent-CPS)

**Fig. 18:**
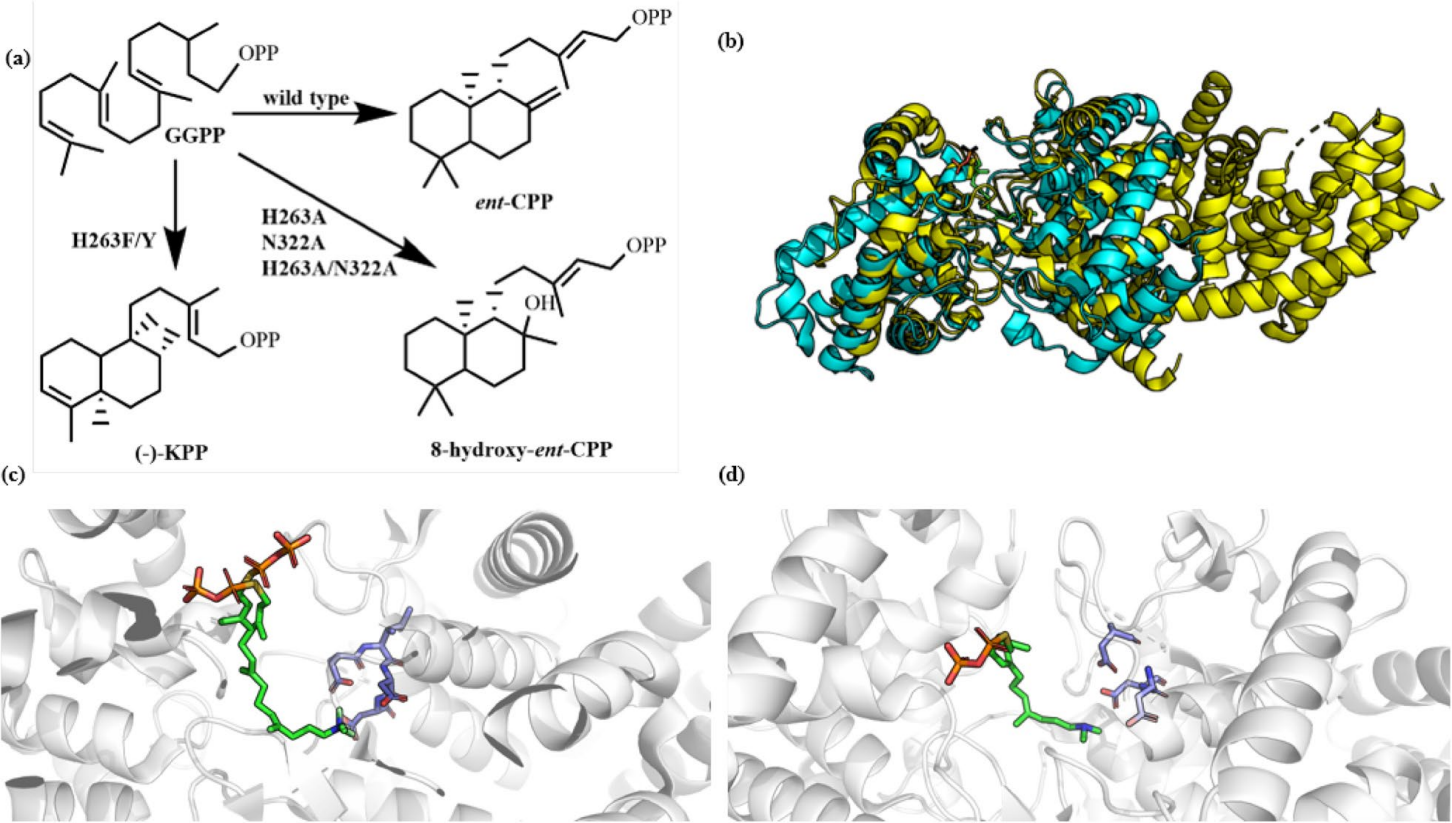
**a** Products of *At*CPS and its mutants. **b** Superposition of *ent*-copalyl diphosphate synthase from *Arabidopsis thaliana* (*At*CPS) in complex with (*S*)-15-aza-14,15-dihydrogeranylgeranyl thiolodiphosphate (PDB ID: **4LIX**, yellow) and *ent*-copalyl diphosphate synthase from *Streptomyces platensis* (*Sp*CPS) (PDB ID: **5BP8**, blue). **c** Active sites of *At*CPS in complex with (*S*)-15-aza-14,15-dihydrogeranylgeranyl thiolodiphosphate (PDB ID: **3PYA**). **d** Active sites of *At*CPS in complex with (*S*)-15-aza-14,15-dihydrogeranylgeranyl thiolodiphosphate (PDB ID: **4LIX**). The substrate analogues and DXDD motif are shown in sticks

*Enantimeric* copalyl diphosphate (*ent*-CPP, Fig. 18a) is an essential precursor of gibberellin (Zi et al. 2014). *At*CPS from *A. thaliana* is the first class II diterpene cyclase with crystal structure analysis. The enantiomer copalyl pyrophosphate synthase (*At*CPS) derived from *A. thaliana* is the first class II diterpene cyclase reported with a crystal structure. This structure is that of the enzyme co-crystallized with a substrate analog, (*S*)-15-aza-14,15-dihydrogeranylgeranyl thiolodiphosphate (PDB ID: **3PYA**) or with a product analogue, 13-aza-13,14-dihydrocopalyl diphosphate (PDB ID: **3PYB**), with resolutions of 2.25 Å and 2.75 Å, respectively (Koeksal et al. 2011). *At*CPS activates the reaction through the protonation of carbon–carbon double bonds promoted by an aspartic acid residue. It has the characteristic sequence of D^377^IDD. The enzyme has 3 domains (α, β, γ), with the active center located at the interface of β and γ domains. The reaction finally achieves the elimination of carbocations by deprotonation.

D379 in the characteristic sequence of D^377^IDD protonates the carbon double bond far away from the pyrophosphate group to initiate the cyclization cascade reaction step, while N425 plays mainly a stabilizing role. It was found that the residue of N332 is conserved, while W369 and W505 may stabilize the carbonium ion in the cyclization process through cation–π interactions; the water molecules in the active center also play an important role in the catalysis; however, in *At*CPS, the metal binding sequence E^199^DEND is far away from the pyrophosphate group in the substrate analogues, which may be due to the fact that Mn^2+^ used in crystallization cannot completely replace Mg^2+^, or the pH value of the system is low, weakening the binding effect of metal ions (Koeksal et al. 2011). Due to the low resolution, it is difficult to analyze the structure–function relationship. Therefore, by optimizing the crystallization conditions and introducing Mg^2+^, a crystal structure with higher resolution was finally obtained (PDB ID: **4LIX**, Fig. 18b). In the previous crystal structure, the pyrophosphate group had two orientations (Fig. 18c), while in the optimized structure (Fig. 18d) the pyrophosphate group has only one single orientation, which explains the positioning effect of Mg^2+^-pyrophosphate group. However, due to the low pH under the crystallization conditions, which affects the binding of metal ions, the crystal structure of Mg^2+^-bound enzyme was not obtained (Koksal et al. 2014). Independent of the substrate channel in the enzyme, there is also a hydrogen ion channel composed of multiple polar amino acids and water molecules, which is conducive to the protonation of carbon–carbon double bonds by D379 (Koksal et al. 2014).

The carbocation in the *At*CPS-catalyzed reaction is finally eliminated by deprotonation to form a double bond, and water molecules act as a generalized base in this step, forming hydrogen bonds with H263 and N322 (Potter et al. 2014). Through site-directed mutagenesis, the resultant mutants H263A, N322A and H263A/N322A were found to produce 8-hydroxy-*ent*-CPP (Fig. 18a), indicating that H263 and N322 play a role in positioning water molecules. After they are mutated to Ala, the hydrogen bonding around water molecules is reduced, making the water molecules directly contact with carbocations to produce hydroxylated products. When H263 and N322 were substituted with aromatic amino acids, it was found that the steps of the cyclization cascade of mutants H263F and H263Y were changed: (−)-kolavenyl diphosphate [(−)-KPP] was formed through hydrogen transfer and methyl transfer (Fig. 18a) (Potter et al. 2016). Szymczyk et al. (2020) investigated the properties of *At*CPS mutants by computer-aided methods, and successfully identified 6 single-site mutants. After combination of the 6 single-site mutations, better mutant enzymes were generated with improved stability and ligand affinity, which enriches the enzyme library of *At*CPS mutants and reveals the key amino acid residues affecting the stability and ligand affinity.

Rudolf et al. (2016) solved the structure of *ent*-CPS derived from *Streptomyces platensis* CB00739 (*Sp*CPS, PDB ID: **5BP8**, Fig. 18b), which is the first class II diterpene cyclase structure derived from bacteria. *Sp*CPS is discovered from the biosynthesis pathway of platensimycin (PTM) and platencin (PTN), but unlike the *At*CPS originating from a plant source, *Sp*CPS contains only two domains, β and γ, without alpha domain. This discovery enriches the knowledge concerning the structure, catalytic mechanism and evolutionary relationship of bacterial diterpene synthases.

Copalyl diphosphate (CPP) has many configurations, including CPP, *ent*-CPP, syn-CPP, syn-ent-CPP which can couple with different terpene synthases to form diverse labdane-related diterpenoids. Reuben J. Peters et al. has done a good summary of the related work (Peters 2010; Zi et al. 2014). These results show that more complex ring structures will be produced when class I and II terpene cyclases are combined to catalyze the formation of cyclized molecules, which suggests that terpene synthases have a rich product library and the ability to form new molecules.

### ***Squalene***–***hopene cyclases***

**Fig. 19:**
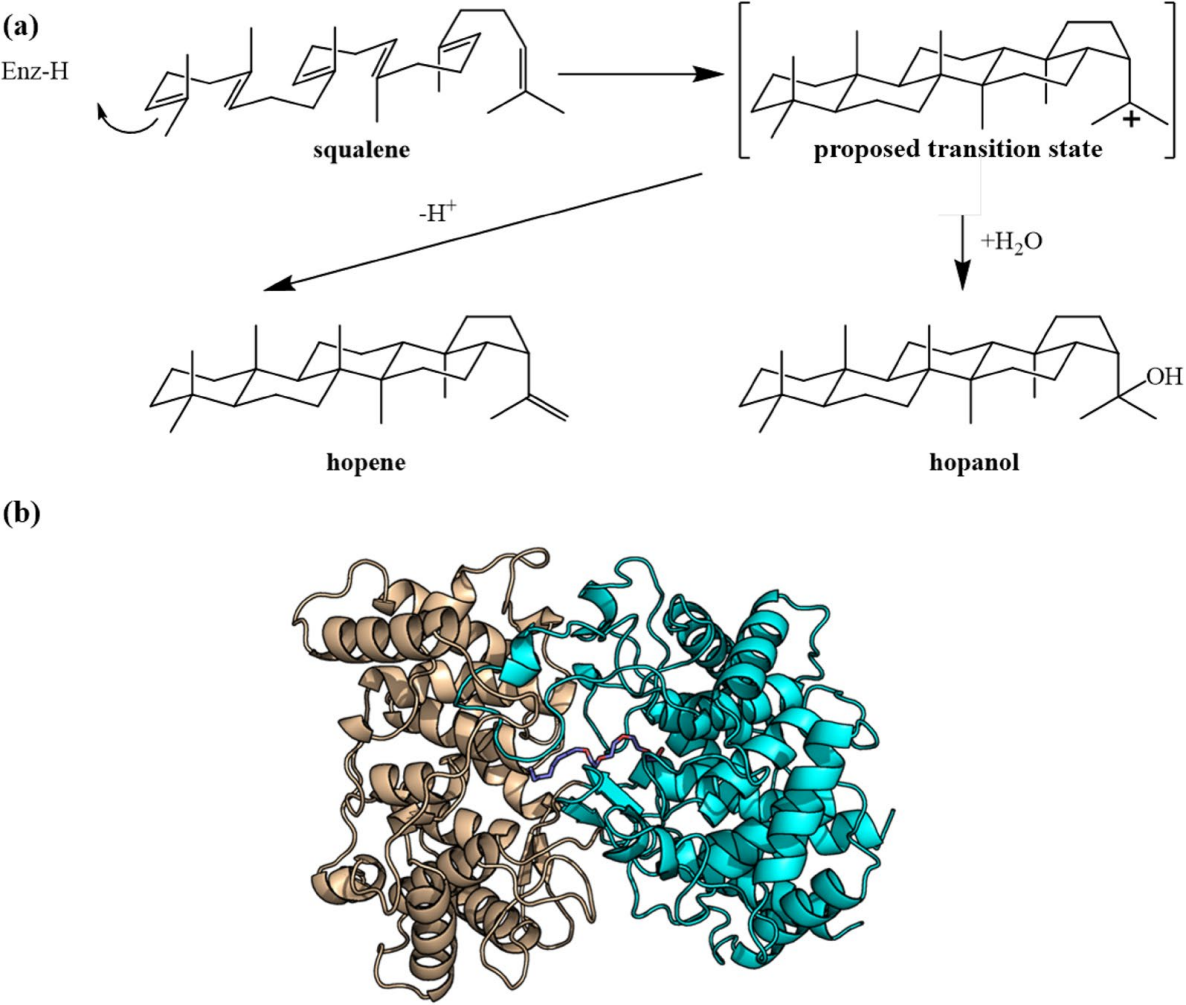
**a** Reaction catalyzed by SHC. **b** Structure of SHC

Squalene–hopene cyclase (SHC) is a triterpene synthase that catalyzes linear isoprenoid precursor squalene in a cyclization cascade to generate hopene (Fig. 19a). Hopene and related triterpene hopanoids influence membrane fluidity (Saenz et al. 2015; Siedenburg and Jendrossek 2011) and pH homeostasis (Welander et al. 2009).

The crystal structure (Fig. 19b) of SHC was first resolved in 1997 with 2.9 Å from *Alicyclobacillus acidocaldarius* (Wendt et al. 1997) and the resolution of the structure was later extended to 2.0 Å as facilitated by a new crystal form (Wendt et al. 1999). The enzyme contains eight so-called QW-sequence repeats, while Oxidosqualene cyclase has five QW-sequence. QW-sequence has been suggested that they shield the cyclases against the released enthalpy of the highly exergonic reaction (Wendt et al. 1999, 1997). SHCs are capable of activating different functionalities other than the traditional terminal isoprene C=C group as well as being compatible with a wide range of nucleophiles beyond the ‘ene-functionality’. Thus, squalene–hopene cyclases demonstrate a great potential to be used as a toolbox for general Brønsted acid catalysis (Hammer et al. 2013). For example, Eichhorn et al. (2018) gained mutants of *A. acidocaldarius* SHC, which are responsible for improved (*E,E*)-homofarnesol conversion. After optimation for reaction condition, A whole cell biotransformation process is presented in which *E. coli* cells producing an improved SHC variant allows the conversion of 125 g/L (*E*,*E*)-homofarnesol to (−)-Ambrox in 72 h. Besides, SHC can product many novel cyclic molecules from different substrates via protein engineering or other methods, which exhibit the promiscuity and application prospect of SHC (Bastian et al. 2017; Eichhorn et al. 2018; Fukuda et al. 2018; Hammer et al. 2015; Ideno et al. 2018; Kuhnel et al. 2017; Nakano et al. 2019; Siedenburg et al. 2013).

## Bifunctional terpene cyclases

Bifunctional terpene synthases which contain two active site of terpene synthases. For example, abietadiene synthase, geosmin synthase (class I–class I), abietadiene synthase (class I–class II), and fusicoccadiene synthase (class I–class I). Here, we pick abietadiene as a typical sample to introduce the structure and function of bifunctional terpene synthases.

### Abietadiene synthase

**Fig. 20:**
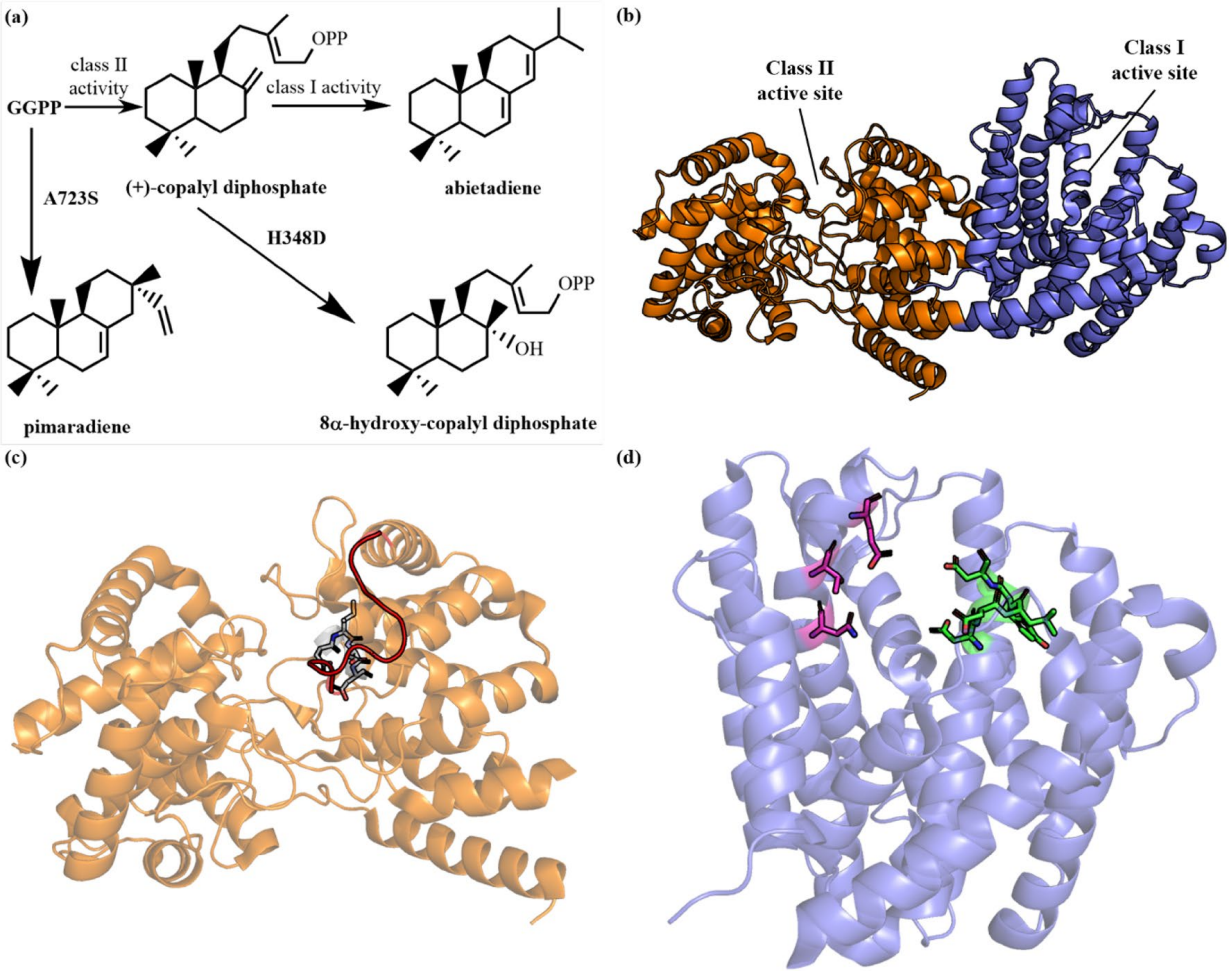
**a** Products of *Ag*AS and its mutants. **b** Crystal structure of *Ag*AS (PDB ID: **3S9V**). **c** Class II active site of *Ag*AS. The motif of class II terpene cyclase (D^402^XDD) are shown in sticks (white). The loop 482–492 is in red. **d** Class I active site of *Ag*AS. The metal binding motifs of class I terpene cyclase: D^621^DXXD, green; N^765^TE, magenta

Abietadiene synthase is a bifunctional terpene cyclase that contains both the active sites of class I and class II cyclases. It can catalyze different reactions and achieve a two-step cyclization process to produce abietadiene (Fig. 20a). Abietadiene synthase (*Ag*AS) from *A. grandis*, is the first bifunctional terpene cyclase (PDB ID: **3S9V**) (Zhou et al. 2012) reported with a crystal structure, possessing 3 domains of α–β–γ (Fig. 20b). Among them, the α domain contains the DDXXD and NTE metal binding motifs of class I terpene cyclase (Fig. 20c), while the β–γ domains contain the metal binding motif (DXDD) of class II terpene cyclase (Fig. 20d). The substrate GGPP is first cyclized within the β–γ domains, forming (+)-copalyl diphosphate [(+)-CPP] (Peters and Croteau 2002; Peters et al. 2001), which diffuses freely into the solvent, and then (+)-CPP binds with the α domain of *Ag*AS (Peters et al. 2001), enabling further cyclization into the final product, abietadiene (Ravn et al. 2000).

N^451^ plays a role in catalyzing the protonation of the carbon–carbon double bond in the functional domain of class II terpene cyclases. N451A mutation showed that *K*_M_ and *k*_cat_ values of the resultant class II cyclase mutant were significantly reduced when compared with the wild type, while the active site of class I cyclase is not significantly affected. Through molecular dynamics simulations, studies on the active center of the class II cyclase of *Ag*AS show that the structural difference from other class II cyclases is mainly located at loop 482–492 (Fig. 20c), which impacts on the GGPP conformation (Zhou et al. 2012).

This protein can produce other circularized molecules through protein engineering (Fig. 20a). For example, the A723S mutant converts abietadiene synthase to pimaradiene cyclase (Wilderman and Peters 2007). H348 acts as a catalytic base to deprotonate the carbocation to form a double bond, producing (+)-CPP, while the H348D mutant eliminates the final carbocations by water molecules, producing 8α-hydroxy-CPP (Schalk et al. 2012).

In 2002, Brodelius et al. (2002) constructed a fusion protein of farnesyl diphosphate synthase and EAS, which produced an artificial bifunctional cyclase converting FPP into *epi*-aristolochene more efficiently. In recent years, the crystal structures of fusicoccadiene synthase (PDB ID: **5ERN**) from *P. amygdali* (Chen et al. 2016b) and geosmin synthase (PDB ID: **5DZ2**) from *S. coelicolor* (Harris et al. 2015) have been resolved, making the knowledge of bifunctional cyclases more abundant. If we can conduct in-depth structure–activity relationship analysis based on the structure of the bifunctional enzyme, it may be possible to create more bifunctional cyclases and to further improve the catalytic efficiency of terpene cyclases.

## Conclusion

Terpene cyclases can cyclize GPP, FPP, GGPP and other linear substrates with only three or less structural functional domains of α, β, and γ, forming monoterpenes, sesquiterpenes, diterpenes and other products with different structures. This shows the terpene cyclase’s ability to precisely control the cyclization reaction. This regulation helps the processes of carbocation generation, transfer and elimination of substrate, which is mainly affected by the contour of the cyclase activity pocket and some aromatic residues or polarities that can interact with carbocations. The elimination of carbocations heralds the end of the entire cyclization reaction. There are two main elimination methods. One is that adjacent carbon atoms deprotonate in some polar residues and/or pyrophosphate groups eliminate carbocations, giving olefin products (Hyatt et al. 2007; Morehouse et al. 2017); another way is to form hydroxyl products with residual water molecules in the active pockets (Croteau et al. 1994; Li et al. 2013) or react with other groups to achieve elimination (Whittington et al. 2002). These two different carbocation elimination methods further increase the molecular diversity of the terpene cyclase catalyzed reactions. By mutating the key residues that stabilize the carbocation, or replacing the hydrophobic amino acid residues in the active pocket, the cyclization reaction can be terminated or even rebuilt to obtain more cyclization products, or to enhance the specificity and catalytic efficiency of the enzyme toward the targeted product.

When the enzyme catalyzes the cyclization reaction, the conformation will change from open to closed state. This conformational change eliminates a large number of water molecules in the active pocket, so that the cyclization process can proceed smoothly, but this does not mean that all water molecules are excluded completely. There are still some water molecules retained, which are essential in maintaining the hydrogen bond network of the active pocket, and have important impacts on the formation of product (Christianson 2017; Kampranis et al. 2007; Koksal et al. 2014; Li et al. 2013; Van Rijn et al. 2019; Whittington et al. 2002). Terpene cyclases have relatively low *K*_M_ values, showing strong affinity with the substrates, but their extremely low *k*_cat_ values also severely limit the efficient production of terpenes (Table 1), indicating a new direction and a great challenge for protein engineering of these terpene cyclases. Both the "role of water" and "catalytic reaction with low kcat " required extensive studies of the exact enzymatic catalytic mechanism at the atomic level, which could be achieved by some promising technologies, such as the isotope labeling experiment and various computational modelling especially the QM/MM simulations.

In this review, the methods for genomic mining of terpene cyclases were classified and sorted out, and several typical crystal structures of cyclases were emphatically introduced. According to the current research, there are more studies regarding the cyclases of monoterpenes and sesquiterpenes, but less are available for those of diterpenoids. With the increase of isoprene units, it is bound to require more precise regulation of the substrate by the cyclase. To strengthen the study on the cyclization enzymes of diterpenes and more complex or active terpenes, it is expected to further elucidate the regulation mechanism of enzymes on terpene cyclization. In recent years, aided with computational biology technologies, the methods for studying the structure and mechanism of cyclization enzymes become more diverse (Freud et al. 2017; Muangphrom et al. 2019; Szymczyk et al. 2020; Van Rijn et al. 2019). It is expected that more enzyme sources with novel structures and properties will be discovered, and more active terpenoid molecules will be synthesized through extensive or intensive exploration of these cyclases. There are some terpene synthases can accept non-natural substrate analogues, such as SHC “Squalene-hopene cyclases” section). These fantastic function attracts chemist to use them as catalysts. Harms et al. (2020) had shed light on the recent advances of these attractive terpene synthase that can catalyze non-natural substrates. This suggested that the chemical diversity of terpenoids still has a great potential to further develop. It is believed that the future research will continue to reveal the diversity and magic of terpene synthases’ structure and function, which plays an increasingly significant role in the production of terpenoids by synthetic biology methods.

## Data Availability

Not applicable.

## Abbreviations

TCs: Terpene cyclases
TSs: Terpene synthases
IPP: Isopentenyl diphosphate
DMAPP: Dimethylallyl diphosphate
MEP: 2-Methyl-d-erythritol-4-phosphate
MVA: Mevalonate acid
GPP: Geranyl pyrophosphate or geranyl diphosphate
FPP: Farnesyl pyrophosphate or farnesyl diphosphate
GGPP: Geranylgeranyl pyrophosphate or geranylgeranyl diphosphate
TPSs: Terpene synthases
OPP: Pyrophosphate group
CPP: Copalyl pyrophosphate or copalyl diphosphate
RACE: Rapid amplification of cDNA ends
As-Ses TPS: Sesquiterpene synthase from *Aquilaria sinensis*
RT-PCR: Reverse transcription-polymerase chain reaction
OSCs: Oxidosqualene cyclases
MeJA: Methyl jasmonate
BPPS: Bornyl diphosphate synthase
BPP: (+)-Bornyl diphosphate
LS: Limonene synthase
LPP: (3*R*)-Linalyl diphosphate
DFGPP: 8,9-Difluorogerany diphosphate
CS: 1,8-Cineole synthase
AS: Aristolochene synthase
SdS: Selinadiene synthase
LinS: Linalool/nerolidol synthase
α-BS: α-Bisabolol synthase
ADS: Amorphadiene synthase
BOeS: α-Bisabolene synthase
EIZS: *epi*-Isozizaene synthase
BTAC: Benzyltriethylammonium cation
MD: Molecular dynamics
EAS: *epi*-Aristolochene synthase
EAH: 5-*epi*-Aristolochene hydroxylase
FDA: U.S. Food and Drug Administration
TS: Taxadiene synthase
ACP: 13-Aza-13,14-dihydrocopalyl diphosphate
FGP: 2-Fluorogeranylgeranyl diphosphate
CotB2: Cyclooctat-9-en-7-ol synthase
*Sm*TS1: Sesterterpene synthase from *Streptomyces mobaraensis*
GFPPS: Geranylfarnesyl diphosphate synthase
GFPP: Geranylfarnesyl diphosphate
StTS: Sesterterpene synthase
PT: Prenyltransferase
CPS: *ent*-Copalyl diphosphate synthase
PTM: Platensimycin
PTN: Platencin
SHC: Squalene-hopene cyclase
*Ag*AS: Abietadiene synthase from *Abies grandis*

## References

[CR1] Aaron JA, Christianson DW (2010). Trinuclear metal clusters in catalysis by terpenoid synthases. Pure Appl Chem.

[CR2] Aaron JA, Lin X, Cane DE, Christianson DW (2010). Structure of *epi*-isozizaene synthase from *Streptomyces coelicolor* A3(2), a platform for new terpenoid cyclization templates. Biochemistry.

[CR3] Alves Gomes Albertti L, Delatte TL, Souza de Farias K, Galdi Boaretto A, Verstappen F, van Houwelingen A, Cankar K, Carollo CA, Bouwmeester HJ, Beekwilder J (2018). Identification of the bisabolol synthase in the endangered Candeia tree (*Eremanthus erythropappus* (DC) McLeisch.). Front Plant Sci.

[CR4] Ansbacher T, Freud Y, Major DT (2018). Slow-starter enzymes: role of active-site architecture in the catalytic control of the biosynthesis of taxadiene by taxadiene synthase. Biochemistry.

[CR5] Attia M, Kim SU, Ro DK (2012). Molecular cloning and characterization of (+)-*epi*-α-bisabolol synthase, catalyzing the first step in the biosynthesis of the natural sweetener, hernandulcin *Lippia Dulcis*. Arch Biochem Biophys.

[CR6] Baer P, Rabe P, Fischer K, Citron CA, Klapschinski TA, Groll M, Dickschat JS (2014). Induced-fit mechanism in class I terpene cyclases. Angew Chem Int Ed Engl.

[CR7] Bastian SA, Hammer SC, Kreß N, Nestl BM, Hauer B (2017). Selectivity in the cyclization of citronellal introduced by squalene hopene cyclase variants. ChemCatChem.

[CR8] Basyuni M, Oku H, Tsujimoto E, Kinjo K, Baba S, Takara K (2007). Triterpene synthases from the Okinawan mangrove tribe. Rhizophoraceae Febs J.

[CR9] Beller HR, Lee TS, Katz L (2015). Natural products as biofuels and bio-based chemicals: fatty acids and isoprenoids. Nat Prod Rep.

[CR10] Bills GF, Gloer JB (2016). Biologically active secondary metabolites from the fungi. Microbiol Spectr.

[CR11] Blank PN, Barrow GH, Chou WKW, Duan L, Cane DE, Christianson DW (2017). Substitution of aromatic residues with polar residues in the active site pocket of *epi*-isozizaene synthase leads to the generation of new cyclic sesquiterpenes. Biochemistry.

[CR12] Blank PN, Barrow GH, Christianson DW (2019). Crystal structure of F95Q *epi*-isozizaene synthase, an engineered sesquiterpene cyclase that generates biofuel precursors β- and γ-curcumene. J Struct Biol.

[CR13] Boutanaev AM, Moses T, Zi J, Nelson DR, Mugford ST, Peters RJ, Osbourn A (2015). Investigation of terpene diversification across multiple sequenced plant genomes. Proc Natl Acad Sci U S A.

[CR14] Brehm-Stecher BF, Johnson EA (2003). Sensitization of *Staphylococcus aureus* and *Escherichia coli* to antibiotics by the sesquiterpenoids nerolidol, farnesol, bisabolol, and apritone. Antimicrob Agents Chemother.

[CR15] Brodelius M, Lundgren A, Mercke P, Brodelius PE (2002). Fusion of farnesyldiphosphate synthase and *epi*-aristolochene synthase, a sesquiterpene cyclase involved in capsidiol biosynthesis in *Nicotiana tabacum*. Eur J Biochem.

[CR16] Calvert MJ, Taylor SE, Allemann RK (2002). Tyrosine 92 of aristolochene synthase directs cyclisation of farnesyl pyrophosphate. Chem Commun (camb).

[CR17] Cane DE, Kang I (2000). Aristolochene synthase: purification, molecular cloning, high-level expression in *Escherichia coli*, and characterization of the *Aspergillus terreus* cyclase. Arch Biochem Biophys.

[CR18] Caruthers JM, Kang I, Rynkiewicz MJ, Cane DE, Christianson DW (2000). Crystal structure determination of aristolochene synthase from the blue cheese mold *Penicillium Roqueforti*. J Biol Chem.

[CR19] Chen M, Al-lami N, Janvier M, D'Antonio EL, Faraldos JA, Cane DE, Allemann RK, Christianson DW (2013). Mechanistic insights from the binding of substrate and carbocation intermediate analogues to aristolochene synthase. Biochemistry.

[CR20] Chen M, Chou WK, Al-Lami N, Faraldos JA, Allemann RK, Cane DE, Christianson DW (2016). Probing the role of active site water in the sesquiterpene cyclization reaction catalyzed by aristolochene synthase. Biochemistry.

[CR21] Chen M, Chou WK, Toyomasu T, Cane DE, Christianson DW (2016). Structure and function of fusicoccadiene synthase, a hexameric bifunctional diterpene synthase. ACS Chem Biol.

[CR22] Chen C-C, Malwal SR, Han X, Liu W, Ma L, Zhai C, Dai L, Huang J-W, Shillo A, Desai J, Ma X, Zhang Y, Guo R-T, Oldfield E (2020). Terpene cyclases and prenyltransferases: Structures and mechanisms of action. ACS Catal.

[CR23] Chen Q, Li J, Liu Z, Mitsuhashi T, Zhang Y, Liu H, Ma Y, He J, Shinada T, Sato T, Wang Y, Liu H, Abe I, Zhang P, Wang G (2020). Molecular basis for sesterterpene diversity produced by plant terpene synthases. Plant Commun.

[CR24] Chiba R, Minami A, Gomi K, Oikawa H (2013). Identification of ophiobolin f synthase by a genome mining approach: a sesterterpene synthase from *Aspergillus clavatus*. Org Lett.

[CR25] Christianson DW (2017). Structural and chemical biology of terpenoid cyclases. Chem Rev.

[CR26] Croteau R, Karp F (1979). Biosynthesis of monoterpenes: preliminary characterization of bornyl pyrophosphate synthetase from sage (*Salvia officinalis*) and demonstration that geranyl pyrophosphate is the preferred substrate for cyclization. Arch Biochem Biophys.

[CR27] Croteau R, Alonso WR, Koepp AE, Johnson MA (1994). Biosynthesis of monoterpenes: partial purification, characterization, and mechanism of action of 1,8-cineole synthase. Arch Biochem Biophys.

[CR28] Daletos G, Katsimpouras C, Stephanopoulos G (2020). Novel strategies and platforms for industrial isoprenoid engineering. Trends Biotechnol.

[CR29] Das S, Shimshi M, Raz K, Nitoker Eliaz N, Mhashal AR, Ansbacher T, Major DT (2019). EnzyDock: protein-ligand docking of multiple reactive states along a reaction coordinate in enzymes. J Chem Theory Comput.

[CR30] Davis EM, Croteau R, Leeper FJ, Vederas JC (2000). Cyclization enzymes in the biosynthesis of monoterpenes, sesquiterpenes, and diterpenes. Biosynthesis: aromatic polyketides, isoprenoids, alkaloids.

[CR31] Despinasse Y, Fiorucci S, Antonczak S, Moja S, Bony A, Nicole F, Baudino S, Magnard JL, Jullien F (2017). Bornyl-diphosphate synthase from *Lavandula angustifolia*: a major monoterpene synthase involved in essential oil quality. Phytochemistry.

[CR32] Dickschat JS (2016). Bacterial terpene cyclases. Nat Prod Rep.

[CR33] Driller R, Janke S, Fuchs M, Warner E, Mhashal AR, Major DT, Christmann M, Bruck T, Loll B (2018). Towards a comprehensive understanding of the structural dynamics of a bacterial diterpene synthase during catalysis. Nat Commun.

[CR34] Driller R, Garbe D, Mehlmer N, Fuchs M, Raz K, Major DT, Bruck T, Loll B (2019). Current understanding and biotechnological application of the bacterial diterpene synthase CotB2. Beilstein J Org Chem.

[CR35] Edgar S, Li FS, Qiao KJ, Weng JK, Stephanopoulos G (2017). Engineering of taxadiene synthase for improved selectivity and yield of a key taxol biosynthetic intermediate. ACS Synth Biol.

[CR36] Eichhorn E, Locher E, Guillemer S, Wahler D, Fourage L, Schilling B (2018). Biocatalytic process for (-)-ambrox production using squalene hopene cyclase. Adv Synth Catal.

[CR37] Fahim M, Ibrahim M, Zahiruddin S, Parveen R, Khan W, Ahmad S, Shrivastava B, Shrivastava AK (2019). TLC-bioautography identification and GC-MS analysis of antimicrobial and antioxidant active compounds in *Musa* x *paradisiaca* L. fruit pulp essential oil. Phytochem Anal.

[CR38] Faraldos JA, Antonczak AK, Gonzalez V, Fullerton R, Tippmann EM, Allemann RK (2011). Probing eudesmane cation-π interactions in catalysis by aristolochene synthase with non-canonical amino acids. J Am Chem Soc.

[CR39] Faraldos JA, Gonzalez V, Senske M, Allemann RK (2011). Templating effects in aristolochene synthase catalysis: elimination *versus* cyclisation. Org Biomol Chem.

[CR40] Faraldos JA, Gonzalez V, Allemann RK (2012). The role of aristolochene synthase in diphosphate activation. Chem Commun (camb).

[CR41] Faraldos JA, Grundy DJ, Cascon O, Leoni S, van der Kamp MW, Allemann RK (2016). Enzymatic synthesis of natural (+)-aristolochene from a non-natural substrate. Chem Commun (camb).

[CR42] Felicetti B, Cane DE (2004). Aristolochene synthase: mechanistic analysis of active site residues by site-directed mutagenesis. J Am Chem Soc.

[CR43] Forcat S, Allemann RK (2006). Stabilisation of transition states prior to and following eudesmane cation in aristolochene synthase. Org Biomol Chem.

[CR44] Freud Y, Ansbacher T, Major DT (2017). Catalytic control in the facile proton transfer in taxadiene synthase. Acs Catal.

[CR45] Fukuda Y, Watanabe T, Hoshino T (2018). Mutated variants of squalene-hopene cyclase: enzymatic syntheses of triterpenes bearing oxygen-bridged monocycles and a new 6,6,6,6,6-fusded pentacyclic scaffold, named neogammacerane, from 2,3-oxidosqualene. Org Biomol Chem.

[CR46] Gao Y, Honzatko RB, Peters RJ (2012). Terpenoid synthase structures: a so far incomplete view of complex catalysis. Nat Prod Rep.

[CR47] Gennadios HA, Gonzalez V, Di Costanzo L, Li A, Yu F, Miller DJ, Allemann RK, Christianson DW (2009). Crystal structure of (+)-δ-cadinene synthase from *Gossypium arboreum* and evolutionary divergence of metal binding motifs for catalysis. Biochemistry.

[CR48] Goncalves MFB, Cardoso SPD, Ferreira UMJ (2020). Overcoming multidrug resistance: Flavonoid and terpenoid nitrogen-containing derivatives as abc transporter modulators. Molecules (basel, Switzerland).

[CR49] Görner C, Häuslein I, Schrepfer P, Eisenreich W, Brück T (2013). Targeted engineering of cyclooctat-9-en-7-ol synthase: a stereospecific access to two new non-natural fusicoccane-type diterpenes. ChemCatChem.

[CR50] Granger RE, Campbell EL, Johnston GA (2005). (+)- And (-)-borneol: efficacious positive modulators of GABA action at human recombinant α_1_β_2_γ_2L_ GABA_A_ receptors. Biochem Pharmacol.

[CR51] Guo K, Liu Y, Li SH (2021) The untapped potential of plant sesterterpenoids: chemistry, biological activities and biosynthesis. Nat Prod Rep. 10.1039/d1np00021g 10.1039/d1np00021g 34114591

[CR52] Hammer SC, Syren P-O, Seitz M, Nestl BM, Hauer B (2013). Squalene hopene cyclases: highly promiscuous and evolvable catalysts for stereoselective C-C and C-X bond formation. Curr Opin Chem Biol.

[CR53] Hammer SC, Marjanovic A, Dominicus JM, Nestl BM, Hauer B (2015). Squalene hopene cyclases are protonases for stereoselective Bronsted acid catalysis. Nat Chem Biol.

[CR54] Hampel D, Mosandl A, Wust M (2005). Biosynthesis of mono- and sesquiterpenes in carrot roots and leaves (*Daucus carota* L): metabolic cross talk of cytosolic mevalonate and plastidial methylerythritol phosphate pathways. Phytochemistry.

[CR55] Harms V, Kirschning A, Dickschat JS (2020). Nature-driven approaches to non-natural terpene analogues. Nat Prod Rep.

[CR56] Harris GG, Lombardi PM, Pemberton TA, Matsui T, Weiss TM, Cole KE, Koksal M, Murphy FVT, Vedula LS, Chou WK, Cane DE, Christianson DW (2015). Structural studies of geosmin synthase, a bifunctional sesquiterpene synthase with α domain architecture that catalyzes a unique cyclization-fragmentation reaction sequence. Biochemistry.

[CR57] Helfrich EJN, Lin G-M, Voigt CA, Clardy J (2019). Bacterial terpene biosynthesis: challenges and opportunities for pathway engineering. Beilstein J Org Chem.

[CR58] Hezari M, Lewis NG, Croteau R (1995). Purification and characterization of taxa-4(5),11(12)-diene synthase from pacific yew (*taxus brevifolia*) that catalyzes the first committed step of taxol biosynthesis. Arch Biochem Biophys.

[CR59] Hou A, Dickschat JS (2020). The biosynthetic gene cluster for sestermobaraenes—discovery of a geranylfarnesyl diphosphate synthase and a multiproduct sesterterpene synthase from *Streptomyces mobaraensis*. Angew Chem Int Ed.

[CR60] Huang AC, Kautsar SA, Hong YJ, Medema MH, Bond AD, Tantillo DJ, Osbourn A (2017). Unearthing a sesterterpene biosynthetic repertoire in the *Brassicaceae* through genome mining reveals convergent evolution. Proc Natl Acad Sci U S A.

[CR61] Huang AC, Hong YJ, Bond AD, Tantillo DJ, Osbourn A (2018). Diverged plant terpene synthases reroute the carbocation cyclization path towards the formation of unprecedented 6/11/5 and 6/6/7/5 sesterterpene scaffolds. Angew Chem Int Ed Engl.

[CR62] Hyatt DC, Youn B, Zhao Y, Santhamma B, Coates RM, Croteau RB, Kang CH (2007). Structure of limonene synthase, a simple model for terpenoid cyclase catalysis. Proc Natl Acad Sci USA.

[CR63] Ideno N, Umeyama S, Watanabe T, Nakajima M, Sato T, Hoshino T (2018). *Alicyclobacillus acidocaldarius* squalene-hopene cyclase: the critical role of steric bulk at Ala306 and the first enzymatic synthesis of epoxydammarane from 2,3-oxidosqualene. ChemBioChem.

[CR64] Janke R, Gorner C, Hirte M, Bruck T, Loll B (2014). The first structure of a bacterial diterpene cyclase: CotB2. Acta Crystallogr D Biol Crystallogr.

[CR65] Jennewein S, Long RM, Williams RM, Croteau R (2004). Cytochrome P450 taxadiene 5α-hydroxylase, a mechanistically unusual monooxygenase catalyzing the first oxygenation step of taxol biosynthesis. Chem Biol.

[CR66] Kampranis SC, Ioannidis D, Purvis A, Mahrez W, Ninga E, Katerelos NA, Anssour S, Dunwell JM, Degenhardt J, Makris AM, Goodenough PW, Johnson CB (2007). Rational conversion of substrate and product specificity in a *Salvia* monoterpene synthase: structural insights into the evolution of terpene synthase function. Plant Cell.

[CR67] Karuppiah V, Ranaghan KE, Leferink NGH, Johannissen LO, Shanmugam M, Ni Cheallaigh A, Bennett NJ, Kearsey LJ, Takano E, Gardiner JM, van der Kamp MW, Hay S, Mulholland AJ, Leys D, Scrutton NS (2017). Structural basis of catalysis in the bacterial monoterpene synthases linalool synthase and 1,8-cineole synthase. ACS Catal.

[CR68] Kawaide H, Imai R, Sassa T, Kamiya Y (1997). Ent-kaurene synthase from the fungus *Phaeosphaeria* sp. L487. cDNA isolation, characterization, and bacterial expression of a bifunctional diterpene cyclase in fungal gibberellin biosynthesis. J Biol Chem.

[CR69] Keeling CI, Bohlmann J (2006). Diterpene resin acids in conifers. Phytochemistry.

[CR70] Keeling CI, Weisshaar S, Lin RPC, Bohlmann J (2008). Functional plasticity of paralogous diterpene synthases involved in conifer defense. Proc Natl Acad Sci U S A.

[CR71] Kim S, Jung E, Kim JH, Park YH, Lee J, Park D (2011). Inhibitory effects of (-)-α-bisabolol on LPS-induced inflammatory response in RAW264.7 macrophages. Food Chem Toxicol.

[CR72] Koeksal M, Hu H, Coates RM, Peters RJ, Christianson DW (2011). Structure and mechanism of the diterpene cyclase *ent*-copalyl diphosphate synthase. Nat Chem Biol.

[CR73] Koksal M, Jin YH, Coates RM, Croteau R, Christianson DW (2011). Taxadiene synthase structure and evolution of modular architecture in terpene biosynthesis. Nature.

[CR74] Koksal M, Potter K, Peters RJ, Christianson DW (2014). 1.55Å-resolution structure of ent-copalyl diphosphate synthase and exploration of general acid function by site-directed mutagenesis. Biochim Biophys Acta.

[CR75] Koo HJ, Vickery CR, Xu Y, Louie GV, O'Maille PE, Bowman M, Nartey CM, Burkart MD, Noel JP (2016). Biosynthetic potential of sesquiterpene synthases: product profiles of Egyptian *Henbane premnaspirodiene* synthase and related mutants. J Antibiot (tokyo).

[CR76] Kuhnel LC, Nestl BM, Hauer B (2017). Enzymatic addition of alcohols to terpenes by squalene hopene cyclase variants. ChemBioChem.

[CR77] Kumar RP, Morehouse BR, Matos JO, Malik K, Lin H, Krauss IJ, Oprian DD (2017). Structural characterization of early michaelis complexes in the reaction catalyzed by (+)-limonene synthase from *Citrus sinensis* using fluorinated substrate analogues. Biochemistry.

[CR78] Lange BM, Ahkami A (2013). Metabolic engineering of plant monoterpenes, sesquiterpenes and diterpenes-current status and future opportunities. Plant Biotechnol J.

[CR79] Li K, Gustafson KR (2020). Sesterterpenoids: chemistry, biology, and biosynthesis. Nat Prod Rep.

[CR80] Li JX, Fang X, Zhao Q, Ruan JX, Yang CQ, Wang LJ, Miller DJ, Faraldos JA, Allemann RK, Chen XY, Zhang P (2013). Rational engineering of plasticity residues of sesquiterpene synthases from *Artemisia annua*: product specificity and catalytic efficiency. Biochem J.

[CR81] Li R, Chou WK, Himmelberger JA, Litwin KM, Harris GG, Cane DE, Christianson DW (2014). Reprogramming the chemodiversity of terpenoid cyclization by remolding the active site contour of *epi*-isozizaene synthase. Biochemistry.

[CR82] Li R, Tee CS, Jiang YL, Jiang XY, Venkatesh PN, Sarojam R, Ye J (2015). A terpenoid phytoalexin plays a role in basal defense of *Nicotiana benthamiana* against *Potato virus X*. Sci Rep.

[CR83] Li Y, Lai Y, Wang Y, Liu N, Zhang F, Xu P (2016). 1, 8-Cineol protect against influenza-virus-induced pneumonia in mice. Inflammation.

[CR84] Li Z, Jiang Y, Zhang X, Chang Y, Li S, Zhang X, Zheng S, Geng C, Men P, Ma L, Yang Y, Gao Z, Tang Y-J, Li S (2020). Fragrant venezuelaenes A and B with A 5–5–6–7 tetracyclic skeleton: discovery, biosynthesis, and mechanisms of central catalysts. ACS Catal.

[CR85] Lin X, Hezari M (1996). Mechanism of taxadiene synthase, a diterpene cyclase that catalyzes the first step of taxol. Biochemistry.

[CR86] Liu Y, Wang L, Jung JH, Zhang S (2007). Sesterterpenoids. Nat Prod Rep.

[CR87] Liu WC, Gong T, Zhu P (2016). Advances in exploring alternative taxol sources. RSC Adv.

[CR88] Matsuda Y, Mitsuhashi T, Quan Z, Abe I (2015). Molecular basis for stellatic acid biosynthesis: a genome mining approach for discovery of sesterterpene synthases. Org Lett.

[CR89] Matsuda Y, Mitsuhashi T, Lee S, Hoshino M, Mori T, Okada M, Zhang H, Hayashi F, Fujita M, Abe I (2016). Astellifadiene: structure determination by NMR spectroscopy and crystalline sponge method, and elucidation of its biosynthesis. Angew Chem Int Ed Engl.

[CR90] McAndrew RP, Peralta-Yahya PP, DeGiovanni A, Pereira JH, Hadi MZ, Keasling JD, Adams PD (2011). Structure of a three-domain sesquiterpene synthase: a prospective target for advanced biofuels production. Structure.

[CR91] Meguro A, Motoyoshi Y, Teramoto K, Ueda S, Totsuka Y, Ando Y, Tomita T, Kim SY, Kimura T, Igarashi M, Sawa R, Shinada T, Nishiyama M, Kuzuyama T (2015). An unusual terpene cyclization mechanism involving a carbon-carbon bond rearrangement. Angew Chem Int Ed Engl.

[CR92] Miele M, Mumot AM, Zappa A, Romano P, Ottaggio L (2012). Hazel and other sources of paclitaxel and related compounds. Phytochem Rev.

[CR93] Misra RC, Maiti P, Chanotiya CS, Shanker K, Ghosh S (2014). Methyl jasmonate-elicited transcriptional responses and pentacyclic triterpene biosynthesis in sweet basil. Plant Physiol.

[CR94] Mitsuhashi T, Rinkel J, Okada M, Abe I, Dickschat JS (2017). Mechanistic characterization of two chimeric sesterterpene synthases from penicillium. Chem Eur J.

[CR95] Morehouse BR, Kumar RP, Matos JO, Olsen SN, Entova S, Oprian DD (2017). Functional and structural characterization of a (+)-limonene synthase from *Citrus sinensis*. Biochemistry.

[CR96] Morehouse BR, Kumar RP, Matos JO, Yu Q, Bannister A, Malik K, Temme JS, Krauss IJ, Oprian DD (2019). Direct evidence of an enzyme-generated LPP intermediate in (+)-limonene synthase using a fluorinated GPP substrate analog. ACS Chem Biol.

[CR97] Muangphrom P, Seki H, Suzuki M, Komori A, Nishiwaki M, Mikawa R, Fukushima EO, Muranaka T (2016). Functional analysis of amorpha-4,11-diene synthase (ADS) homologs from non-artemisinin-producing artemisia species: The discovery of novel koidzumiol and (+)-α-bisabolol synthases. Plant Cell Physiol.

[CR98] Muangphrom P, Misaki M, Suzuki M, Shimomura M, Suzuki H, Seki H, Muranaka T (2019). Identification and characterization of (+)-α-bisabolol and 7-*epi*-silphiperfol-5-ene synthases from *Artemisia abrotanum*. Phytochemistry.

[CR99] Muller J, Greiner JF, Zeuner M, Brotzmann V, Schafermann J, Wieters F, Widera D, Sudhoff H, Kaltschmidt B, Kaltschmidt C (2016). 1,8-Cineole potentiates IRF3-mediated antiviral response in human stem cells and in an *ex vivo* model of rhinosinusitis. Clin Sci (lond).

[CR100] Murata Y, Kokuryo T, Yokoyama Y, Yamaguchi J, Miwa T, Shibuya M, Yamamoto Y, Nagino M (2017). The anticancer effects of novel α-bisabolol derivatives against pancreatic cancer. Anticancer Res.

[CR101] Nakano C, Kudo F, Eguchi T, Ohnishi Y (2011). Genome mining reveals two novel bacterial sesquiterpene cyclases: (-)-germacradien-4-ol and (-)-*epi*-α-bisabolol synthases from *Streptomyces citricolor*. ChemBioChem.

[CR102] Nakano C, Watanabe T, Minamino M, Hoshino T (2019). Enzymatic syntheses of novel carbocyclic scaffolds with a 6,5 + 5,5 ring system by squalene-hopene cyclase. Org Biomol Chem.

[CR103] O'Brien TE, Bertolani SJ, Tantillo DJ, Siegel JB (2016). Mechanistically informed predictions of binding modes for carbocation intermediates of a sesquiterpene synthase reaction. Chem Sci.

[CR104] O'Brien TE, Bertolani SJ, Zhang Y, Siegel JB, Tantillo DJ (2018). Predicting productive binding modes for substrates and carbocation intermediates in terpene synthases-bornyl diphosphate synthase as a representative case. ACS Catal.

[CR105] Oikawa H, Toyomasu T, Toshima H, Ohashi S, Kawaide H, Kamiya Y, Ohtsuka M, Shinoda S, Mitsuhashi W, Sassa T (2001). Cloning and functional expression of cDNA encoding Aphidicolan-16 beta-ol synthase: a key enzyme responsible for formation of an unusual diterpene skeleton in biosynthesis of aphidicolin. J Am Chem Soc.

[CR106] Okada M, Matsuda Y, Mitsuhashi T, Hoshino S, Mori T, Nakagawa K, Quan Z, Qin B, Zhang H, Hayashi F, Kawaide H, Abe I (2016). Genome-based discovery of an unprecedented cyclization mode in fungal sesterterpenoid biosynthesis. J Am Chem Soc.

[CR107] O'Maille PE, Malone A, Dellas N, Andes Hess B, Smentek L, Sheehan I, Greenhagen BT, Chappell J, Manning G, Noel JP (2008). Quantitative exploration of the catalytic landscape separating divergent plant sesquiterpene synthases. Nat Chem Biol.

[CR108] Pemberton TA, Chen MB, Harris GG, Chou WKW, Duan L, Koksal M, Genshaft AS, Cane DE, Christianson DW (2017). Exploring the influence of domain architecture on the catalytic function of diterpene synthases. Biochemistry.

[CR109] Peralta-Yahya PP, Ouellet M, Chan R, Mukhopadhyay A, Keasling JD, Lee TS (2011). Identification and microbial production of a terpene-based advanced biofuel. Nat Commun.

[CR110] Peters RJ (2010). Two rings in them all: the labdane-related diterpenoids. Nat Prod Rep.

[CR111] Peters RJ, Croteau RB (2002). Abietadiene synthase catalysis: conserved residues involved in protonation-initiated cyclization of geranylgeranyl diphosphate to (+)-copalyl diphosphate. Biochemistry.

[CR112] Peters RJ, Ravn MM, Coates RM, Croteau RB (2001). Bifunctional abietadiene synthase: free diffusive transfer of the (+)-copalyl diphosphate intermediate between two distinct active sites. J Am Chem Soc.

[CR113] Potter K, Criswell J, Zi J, Stubbs A, Peters RJ (2014). Novel product chemistry from mechanistic analysis of *ent*-copalyl diphosphate synthases from plant hormone biosynthesis. Angew Chem Int Ed Engl.

[CR114] Potter KC, Zi J, Hong YJ, Schulte S, Malchow B, Tantillo DJ, Peters RJ (2016). Blocking deprotonation with retention of aromaticity in a plant *ent*-copalyl diphosphate synthase leads to product rearrangement. Angew Chem Int Ed Engl.

[CR115] Qin B, Matsuda Y, Mori T, Okada M, Quan Z, Mitsuhashi T, Wakimoto T, Abe I (2016). An unusual chimeric diterpene synthase from *Emericella variecolor* and its functional conversion into a sesterterpene synthase by domain swapping. Angew Chem Int Ed Engl.

[CR116] Ravn MM, Coates RM, Flory JE, Peters RJ, Croteau R (2000). Stereochemistry of the cyclization-rearrangement of (+)-copalyl diphosphate to (-)-abietadiene catalyzed by recombinant abietadiene synthase from *Abies grandis*. Org Lett.

[CR117] Raz K, Driller R, Dimos N, Ringel M, Bruck T, Loll B, Major DT (2020). The impression of a nonexisting catalytic effect: the role of CotB2 in guiding the complex biosynthesis of cyclooctat-9-en-7-ol. J Am Chem Soc.

[CR118] Rising KA, Starks CM, Noel JP, Chappell J (2000). Demonstration of germacrene A as an intermediate in 5-*epi*-aristolochene synthase catalysis. J Am Chem Soc.

[CR119] Rising KA, Crenshaw CM, Koo HJ, Subramanian T, Chehade KAH, Starks C, Allen KD, Andres DA, Spielmann HP, Noel JP, Chappell J (2015). Formation of a novel macrocyclic alkaloid from the unnatural farnesyl diphosphate analogue anilinogeranyl diphosphate by 5-*epi*-aristolochene synthase. ACS Chem Biol.

[CR120] Rudolf JD, Dong LB, Cao H, Hatzos-Skintges C, Osipiuk J, Endres M, Chang CY, Ma M, Babnigg G, Joachimiak A, Phillips GN, Shen B (2016). Structure of the *ent*-copalyl diphosphate synthase PtmT2 from *Streptomyces platensis* CB00739, a bacterial type II diterpene synthase. J Am Chem Soc.

[CR121] Rudolph K, Parthier C, Egerer-Sieber C, Geiger D, Muller YA, Kreis W, Muller-Uri F (2016). Expression, crystallization and structure elucidation of γ-terpinene synthase from *Thymus vulgaris*. Acta Crystallogr F Struct Biol Commun.

[CR122] Ruzicka L (1953). The isoprene rule and the biogenesis of terpenic compounds. Experientia.

[CR123] Saenz JP, Grosser D, Bradley AS, Lagny TJ, Lavrynenko O, Broda M, Simons K (2015). Hopanoids as functional analogues of cholesterol in bacterial membranes. Proc Natl Acad Sci U S A.

[CR124] Schalk M, Pastore L, Mirata MA, Khim S, Schouwey M, Deguerry F, Pineda V, Rocci L, Daviet L (2012). Toward a biosynthetic route to sclareol and amber odorants. J Am Chem Soc.

[CR125] Schrepfer P, Buettner A, Goerner C, Hertel M, van Rijn J, Wallrapp F, Eisenreich W, Sieber V, Kourist R, Bruck T (2016). Identification of amino acid networks governing catalysis in the closed complex of class I terpene synthases. Proc Natl Acad Sci USA.

[CR126] Seki T, Kokuryo T, Yokoyama Y, Suzuki H, Itatsu K, Nakagawa A, Mizutani T, Miyake T, Uno M, Yamauchi K, Nagino M (2011). Antitumor effects of α-bisabolol against pancreatic cancer. Cancer Sci.

[CR127] Seol GH, Kim KY (2016). Eucalyptol and its role in chronic diseases. Adv Exp Med Biol.

[CR128] Shao J, Chen QW, Lv HJ, He J, Liu ZF, Lu YN, Liu HL, Wang GD, Wang Y (2017). (+)-thalianatriene and (-)-retigeranin B catalyzed by Sesterterpene synthases from *Arabidopsis thaliana*. Org Lett.

[CR129] Shibuya M, Katsube Y, Otsuka M, Zhang H, Tansakul P, Xiang T, Ebizuka Y (2009). Identification of a product specific β-amyrin synthase from *Arabidopsis thaliana*. Plant Physiol Biochem.

[CR130] Shishova EY, Costanzo LD, Cane DE, Christianson DW (2006). X-ray crystal structure of aristolochene synthase from *Aspergillus terreus* and evolution of templates for the cyclization of farnesyl diphosphate. Biochemistry.

[CR131] Siedenburg G, Jendrossek D (2011). Squalene-hopene cyclases. Appl Environ Microbiol.

[CR132] Siedenburg G, Breuer M, Jendrossek D (2013). Prokaryotic squalene-hopene cyclases can be converted to citronellal cyclases by single amino acid exchange. Appl Microbiol Biotechnol.

[CR133] Silva EAP, Carvalho JS, Guimaraes AG, Barreto RSS, Santos MRV, Barreto AS, Quintans-Junior LJ (2019). The use of terpenes and derivatives as a new perspective for cardiovascular disease treatment: a patent review (2008–2018). Expert Opin Ther Pat.

[CR134] Singh B, Sharma RA (2015). Plant terpenes: defense responses, phylogenetic analysis, regulation and clinical applications. 3 Biotech.

[CR135] Srividya N, Davis EM, Croteau RB, Lange BM (2015). Functional analysis of (4*S*)-limonene synthase mutants reveals determinants of catalytic outcome in a model monoterpene synthase. Proc Natl Acad Sci U S A.

[CR136] Starks CM, Back K, Chappell J, Noel JP (1997). Structural basis for cyclic terpene biosynthesis by tobacco 5-*epi*-aristolochene synthase. Science.

[CR138] Szymczyk P, Szymanska G, Lipert A, Weremczuk-Jezyna I, Kochan E (2020). Computer-aided saturation mutagenesis of *Arabidopsis thaliana ent*-copalyl diphosphate synthase. Interdiscip Sci.

[CR139] Tang X, Zhang F, Zeng T, Li W, Yin S, Wu R (2020). Enzymatic plasticity inspired by the diterpene cyclase CotB2. ACS Chem Biol.

[CR140] Tomita T, Kim SY, Teramoto K, Meguro A, Ozaki T, Yoshida A, Motoyoshi Y, Mori N, Ishigami K, Watanabe H, Nishiyama M, Kuzuyama T (2017). Structural insights into the CotB2-catalyzed cyclization of geranylgeranyl diphosphate to the diterpene cyclooctat-9-en-7-ol. ACS Chem Biol.

[CR141] Toyomasu T, Kawaide H, Ishizaki A, Shinoda S, Otsuka M, Mitsuhashi W, Sassa T (2000). Cloning of a full-length cDNA encoding *ent*-kaurene synthase from *Gibberella fujikuroi*: functional analysis of a bifunctional diterpene cyclase. Biosci Biotechnol Biochem.

[CR142] Toyomasu T, Nuda R, Kenmoku H, Kanno Y, Miura S, Nakano C, Shiono Y, Mitsuhashi W, Toshima H, Oikawa H, Hoshino T, Dairi T, Kato N, Sassa T (2008). Identification of diterpene biosynthetic gene clusters and functional analysis of labdane-related diterpene cyclases in *Phomopsis amygdali*. Biosci Biotechnol Biochem.

[CR143] Valdes M, Calzada F, Mendieta-Wejebe JE, Merlin-Lucas V, Velazquez C, Barbosa E (2020). Antihyperglycemic effects of *Annona diversifolia* Safford and its acyclic terpenoids: α-glucosidase and selective SGLT1 inhibitiors. Molecules (basel, Switzerland).

[CR144] Van der Kamp MW, Sirirak J, Zurek J, Allemann RK, Mulholland AJ (2013). Conformational change and ligand binding in the aristolochene synthase catalytic cycle. Biochemistry.

[CR145] Van Rijn JPM, Escorcia AM, Thiel W (2019). QM/MM study of the taxadiene synthase mechanism. J Comput Chem.

[CR146] Wang L, Yang B, Lin XP, Zhou XF, Liu Y (2013). Sesterterpenoids. Nat Prod Rep.

[CR147] Welander PV, Hunter RC, Zhang L, Sessions AL, Summons RE, Newman DK (2009). Hopanoids play a role in membrane integrity and pH homeostasis in *Rhodopseudomonas palustris* TIE-1. J Bacteriol.

[CR148] Wendt KU, Schulz GE (1998). Isoprenoid biosynthesis: manifold chemistry catalyzed by similar enzymes. Structure (london).

[CR149] Wendt KU, Poralla K, Schulz GE (1997). Structure and function of a squalene cyclase. Science (wash D c).

[CR150] Wendt KU, Lenhart A, Schulz GE (1999). The structure of the membrane protein squalene-hopene cyclase at 2.0 angstrom resolution. J Mol Biol.

[CR151] Whittington DA, Wise ML, Urbansky M, Coates RM, Croteau RB, Christianson DW (2002). Bornyl diphosphate synthase: structure and strategy for carbocation manipulation by a terpenoid cyclase. Proc Natl Acad Sci USA.

[CR152] Wilderman PR, Peters RJ (2007). A single residue switch converts abietadiene synthase into a pimaradiene specific cyclase. J Am Chem Soc.

[CR153] Williams DC, Wildung MR, Jin AQ, Dalal D, Oliver JS, Coates RM, Croteau R (2000). Heterologous expression and characterization of a "Pseudomature" form of taxadiene synthase involved in paclitaxel (Taxol) biosynthesis and evaluation of a potential intermediate and inhibitors of the multistep diterpene cyclization reaction. Arch Biochem Biophys.

[CR154] Withers ST, Gottlieb SS, Lieu B, Newman JD, Keasling JD (2007). Identification of isopentenol biosynthetic genes from Bacillus subtilis by a screening method based on isoprenoid precursor toxicity. Appl Environ Microbiol.

[CR155] Xu Y, Zhang Z, Wang M, Wei J, Chen H, Gao Z, Sui C, Luo H, Zhang X, Yang Y, Meng H, Li W (2013). Identification of genes related to agarwood formation: transcriptome analysis of healthy and wounded tissues of *Aquilaria sinensis*. BMC Genom.

[CR156] Xu J, Ai Y, Wang J, Xu J, Zhang Y, Yang D (2017). Converting *S*-limonene synthase to pinene or phellandrene synthases reveals the plasticity of the active site. Phytochemistry.

[CR157] Xu J, Xu J, Ai Y, Farid RA, Tong L, Yang D (2018). Mutational analysis and dynamic simulation of *S*-limonene synthase reveal the importance of Y573: insight into the cyclization mechanism in monoterpene synthases. Arch Biochem Biophys.

[CR158] Yang CH, Horwitz SB (2017). Taxol®: The first microtubule stabilizing agent. Int J Mol Sci.

[CR159] Yang Y-L, Zhang S, Ma K, Xu Y, Tao Q, Chen Y, Chen J, Guo S, Ren J, Wang W, Tao Y, Yin W-B, Liu H (2017). Discovery and characterization of a new family of diterpene cyclases in bacteria and fungi. Angew Chem Int Ed.

[CR160] Ye Y, Minami A, Mandi A, Liu C, Taniguchi T, Kuzuyama T, Monde K, Gomi K, Oikawa H (2015). Genome mining for Sesterterpenes using bifunctional terpene synthases reveals a unified intermediate of di/sesterterpenes. J Am Chem Soc.

[CR161] Ye W, He X, Wu H, Wang L, Zhang W, Fan Y, Li H, Liu T, Gao X (2018). Identification and characterization of a novel sesquiterpene synthase from *Aquilaria sinensis*: an important gene for agarwood formation. Int J Biol Macromol.

[CR162] Zhang F, Chen N, Wu R (2016). Molecular dynamics simulations elucidate conformational dynamics responsible for the cyclization reaction in TEAS. J Chem Inf Model.

[CR163] Zhang F, Chen N, Zhou J, Wu R (2016). Protonation-dependent diphosphate cleavage in FPP cyclases and synthases. ACS Catal.

[CR164] Zhang Z, Luo Z, Bi A, Yang W, An W, Dong X, Chen R, Yang S, Tang H, Han X, Luo L (2017). Compound edaravone alleviates lipopolysaccharide (LPS)-induced acute lung injury in mice. Eur J Pharmacol.

[CR165] Zhang F, Wang YH, Tang X, Wu R (2018). Catalytic promiscuity of the non-native FPP substrate in the TEAS enzyme: non-negligible flexibility of the carbocation intermediate. Phys Chem Chem Phys.

[CR166] Zhang F, An T, Tang X, Zi J, Luo H-B, Wu R (2019). Enzyme promiscuity versus fidelity in two sesquiterpene cyclases (TEAS versus ATAS). ACS Catal.

[CR167] Zhao B, Lin X, Lei L, Lamb DC, Kelly SL, Waterman MR, Cane DE (2008). Biosynthesis of the sesquiterpene antibiotic albaflavenone in *Streptomyces coelicolor* A3(2). J Biol Chem.

[CR168] Zhou K, Peters RJ (2009). Investigating the conservation pattern of a putative second terpene synthase divalent metal binding motif in plants. Phytochemistry.

[CR169] Zhou K, Gao Y, Hoy JA, Mann FM, Honzatko RB, Peters RJ (2012). Insights into diterpene cyclization from structure of bifunctional abietadiene synthase from *Abies grandis*. J Biol Chem.

[CR170] Zi JC, Mafu S, Peters RJ (2014). To gibberellins and beyond! Surveying the evolution of (di)terpenoid metabolism. Annu Rev Plant Biol.

